# Comparative transcriptome and metabolite profiling of four tissues from *Alisma orientale* (Sam.) Juzep reveals its inflorescence developmental and medicinal characteristics

**DOI:** 10.1038/s41598-019-48806-w

**Published:** 2019-08-23

**Authors:** Wenjin Lin, Fengling Sun, Yamin Zhang, Xiaomei Xu, Xuehua Lu, Lisha Li, Rongqing Xu

**Affiliations:** Fujian Key Laboratory of Medical Measurement, Fujian Academy of Medical Sciences, Fuzhou, China

**Keywords:** Transcriptomics, Plant molecular biology, Secondary metabolism

## Abstract

*Alisma orientale* (Sam.) Juzep (*A*. *orientale*) is an important medicinal plant in traditional Chinese medicine. In this study, *de novo* RNA-seq of *A*. *orientale* was performed based on the cDNA libraries from four different tissues, roots, leaves, scapes and inflorescences. A total of 41,685 unigenes were assembled, 25,024 unigene functional annotations were obtained by searching against the five public sequence databases, and 3,411 simple sequence repeats in *A*. *orientale* were reported for the first time. 15,402 differentially expressed genes were analysed. The morphological characteristics showed that compared to the other tissues, the leaves had more chlorophyll, the scapes had more vascular bundles, and the inflorescences contained more starch granules and protein. In addition, the metabolic profiles of eight kinds of alisols metabolite profiling, which were measured by ultra-Performance liquid chromatography-triple quadrupole-mass spectrometry showed that alisol B 23-acetate and alisol B were the major components of the four tissues at amounts of 0.068~0.350 mg/g and 0.046~0.587 mg/g, respectively. In addition, qRT-PCR validated that farnesyl pyrophosphate synthase and 3-hydroxy-3-methylglutaryl-CoA reductase should be considered the critical candidate genes involved in alisol biosynthesis. These transcriptome and metabolic profiles of *A*. *orientale* may help clarify the molecular mechanisms underlying the medicinal characteristics of *A*. *orientale*.

## Introduction

*Alisma orientale* (Sam.) Juzep (*A*. *orientale*) is a perennial aquatic or marsh herb belonging to the *Alisma* genus and Alismataceae family^1^. The tuber has been used as Chinese materia medica for approximately two thousand years, and it is named *Zexie* in China and *Takusha* in Japanese^2,3^. *A*. *orientale* was first recorded in an herbal monograph *Shennong*’*s Classic of Materia Medica*, that was written during the Han dynasty, and it was classified as an top grade herb due to its low toxicity. On the basis of the Compendium of Materia Medica, *A*. *orientale* could be used to eliminate oedema, as a diuretic, and to reduce fevers^1^. Therefore, it was extensively used to treat various urinary system diseases, such as oedema, gonorrhoea and dizziness in ancient times^4^. Recently, it was proven that *A*. *orientale* contains many active constituents, such as alisol B 23-acetate, alisol B, alisol A 24-acetate, alisol A, 17-epi alisolide, and 24-epi alismanol D^5^. *A*. *orientale *also has therapeutic effects on hyperlipidaemia^6,7^, chronic prostatitis^8^, chronic obstructive pulmonary disease^9^, hepatic steatosis^10^, diabetes^11^, prophylaxis of urolithiasis^12^, anti-atherosclerotic actions^13^, cholestasis^14^, chronic kidney disease^15^ and cancer^16–18^.

*A*. *orientale* is the medicinal source of prepared drug in pieces of Zexie. Since 1977, it has been included in the Pharmacopoeia of the People’s Republic of China. In the Chinese medicinal herbs trade market, *Alisma plantago-aquatica* L.(*A*. *plantago*) is the other important medicinal ingredient of Zexie Decoction^19–22^, and *A*. *plantago* belongs to the genus *Alisma* genus; however, its scape and inflorescence appearance are different from *A*. *orientale*, moreover, the scape and inflorescence of *A*. *orientale* is edible, and its economic value as a vegetable is higher than its value as a medicinal tuber. Currently, the scape and inflorescence of *A*. *plantago* are not suitable for consumption as a vegetable, *A*. *plantago* is cultivated only for producing medicine. According to another herbal monograph titled *Mingyi bielu*, the roots, leaves, and fruits of *A*. *orientale* have also been used as Chinese materia medica after harvesting. However, current studies have concentrated on the tuber of *A*. *orientale*, and none have reported on the scape and inflorescence. Moreover, the large aerial parts and roots of *A*. *orientale* are considered to be of no use and are discarded. To date, only the microstructure of the *A*. *orientale* tuber has been reported, and the morphological characteristics of the roots, leaves, scapes and inflorescences in *A*. *orientale* have not yet been elucidated. Therefore, it is necessary to conduct more medicinal research on the other tissues, especially on the scape and inflorescence of *A*. *orientale*.

Until now, there have been few studies on the molecular mechanisms of the growth, development and secondary metabolism of *A*. *orientale*^23–27^. One of the main bottlenecks of *A*. *orientale* molecular mechanisms researches is that there are no genomic sequences and gene annotations for *A*. *orientale*; therefore, there is not adequate information to study the molecular basis of the development and secondary metabolism of *A*. *orientale*. Currently, RNA-seq is an economical and viable sequencing technique to discover new genes and differential gene expression at the transcription level in various plants, such as rice^28–30^, soybean^31,32^, pear^33–35^, *Brassica napus*^36–39^, *Baphicacanthus cusia*^40^, chrysanthemum^41–43^, and Litchi^44,45^. Therefore, in the absence of the *A*. *orientale* genome, transcriptome sequencing of *A*. *orientale* is an effective and practical way to obtain a large amount of gene information on *A*. *orientale*.

In this study, first, high-throughput sequencing technology was employed to obtain the transcriptomes of four different tissues, root, leaf, scape and inflorescences of *A*. *orientale*. Simple sequence repeat (SSR) markers in *A*. *orientale* were reported for the first time. The unigene annotation and differentially expressed genes (DEGs), especially DEGs involved in the alisol and its derivatives biosynthesis were analysed. Second, research on the morphological characteristics of the roots, leaves, scapes and inflorescences from *A*. *orientale* was performed after paraffin sections stained by iodine-potassium iodide (I-KI) and fast green. Liquid chromatography-mass spectrometry was used to determine the content of eight kinds of alisol and its derivatives in different tissues. Furthermore, qRT-PCR was carried out to validate the expression levels of the DEGs involved in the biosynthesis of alisol and its derivatives and aquaporin-related pathways. We believe that these transcriptome and metabolic profiles of *A*. *orientale* may help elucidate the molecular mechanisms underlying the distribution characteristics of alisol and its derivatives.

## Results

### Microscopic structure characteristics

**Figure 1:**
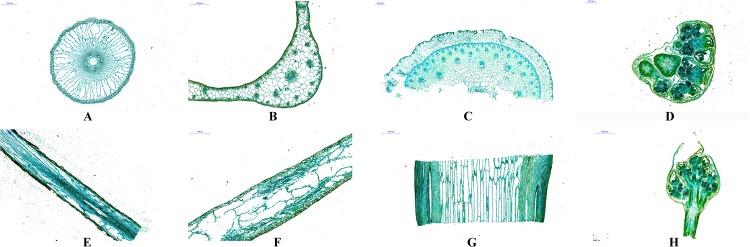
Microstructure structure of the roots, leaves, scapes and inflorescences of *A*. *orientale* under I-KI and fast green exposure. (**A**–**D**) Transverse section of the root (20x), leaf (100x), scape (100x) and inflorescence (50x); (**E**–**H**) vertical section of the root (50x), leaf (50x), scape (100x) and inflorescence (200x). When stained with I-KI, the starch granules are blue or blue-black, and the protein is yellow. When stained with fast green, the cell wall is green.

Optical microscopy revealed the anatomical structure of the roots (RT), leaves (LF), scapes (SC) and inflorescences (IN) from *A*. *orientale* (Figs 1 and S1). The transverse section of the root was round, and the outermost layer was the epidermis, which was partially removed (Fig. 1A,E). The inner side of the epidermis was the cortex, and the outer cortex consisted of three layers of small irregular cells that were closely arranged. The parenchyma cells in the cortex formed a large amount of long irregular aerated tissue, and there was a small amount of starch granules in the cortical parenchyma cells, The cortex was closely packed and the inner lignification thickened to form the Kjeldahl belt. The vascular bundle in the fibrous root was a finite vascular bundle, and the outermost lateral sheath was composed of parenchyma cells, which were regularly arranged. The xylem and phloem were inside the vascular bundle, and no layer was formed. The medulla was circular and located at the innermost side of the vascular bundle.

The leaves were typically two-sided leaves (Fig. 1B,F). The upper epidermis was closely arranged in a series of rectangular cells. The epidermal cells were round, the surface of the leaves was smooth and free of glandular hairs, the stomata were distributed in the upper epidermis, and the number of stomata was slightly higher in the upper epidermis than in the lower epidermis. The mesophyll tissue was divided into two kinds: palisade tissue and sponge tissue. The palisade tissue consisted of two rows of cells, which contained more chloroplasts than did the cells in the sponge tissue and did not pass through the midrib. The sponge tissue was loosely arranged, and the number of chloroplasts in the sponge cells near the epidermis was less than that of the palisade cells. The vascular bundle was limited external toughness, the vascular bundle sheath contained the phloem and xylem, the xylem was arranged in an arc around the pith, and the medullary cavity was small or absent. The chloroplast content in the young leaves was small, and there were noticeable vascular bundles in the main vein and the main branch veins, and there was less ventilating tissue inside the leaves than inside the other plant tissues.

The scape of *A*. *orientale* was leafless peduncle originating from a basal rosette of a stemless plant (Fig. 1C,G). The transverse section of the scape was round, and the outermost layer was the epidermis, which contained 3~6 layers of parenchyma cells. The cortex had 3~5 rounds of vascular bundles arranged in a ring, the distribution of vascular bundles was similar to that in the petiole, and the middle vascular bundle was larger than the other types of vascular bundles. However, there were fewer middle vascular bundles than other kinds of vascular bundles, and close to the epidermis, the vascular bundles were smaller in size and greater in number. The type and structure of the vascular bundles close to the epidermis were the same as those of the vascular bundle in the petiole, and the type of vascular bundles was collateral closed. The vascular bundle in the petiole had a larger medullary cavity, and there were a few starch granules in the parenchyma cells.

The inflorescence grew in the head of the sacpes, were heart-shaped and not mature, and had 3~9 compartments and 3~9 chambers per interval, which contained more starch granules than did other parts of the inflorescence.The membrane contains more protein than did other parts of the inflorescence (Fig. 1D,H).

### RNA-seq and de novo assembly

The RNA-seq data have been submitted to the National Center for Biotechnology Information (NCBI) SRA database with the accession number SRP124598, PRJNA417185. In this study, sequence data of 7.31 Gb for RT, 7.27 Gb for LF, 7.46 Gb for SC and 7.62 Gb for IN were generated. There were 91,962,600 high quality reads for RT, 94,101,458 for LF, 90,311,938 for SC and 96,595,132 for IN. A total of 372,971,128 high-quality reads from the four different tissues were assembled into 41,685 unigenes. There were 40,375 unigenes expressed in RT, 39,231 unigenes expressed in LF, 39,450 unigenes expressed in SC, and 40,174 unigenes expressed in IN, respectively. Among them, 651 unigenes detected only in RT, 27 unigenes detected only in LF, 16 unigenes detected only in SC, 111 unigenes detected only in IN (Fig. S2). They were tissue-specific loci, which may be important in tissue-specific development. These unigene lengths ranged from 199 bp to13,455 bp, the average length was 1,117 bp, the N50 length was 1,727 bp, the GC percentage was 47.39%, and the number of total assembled bases were 46,562,041.

### Unigene functional annotation

**Figure 2:**
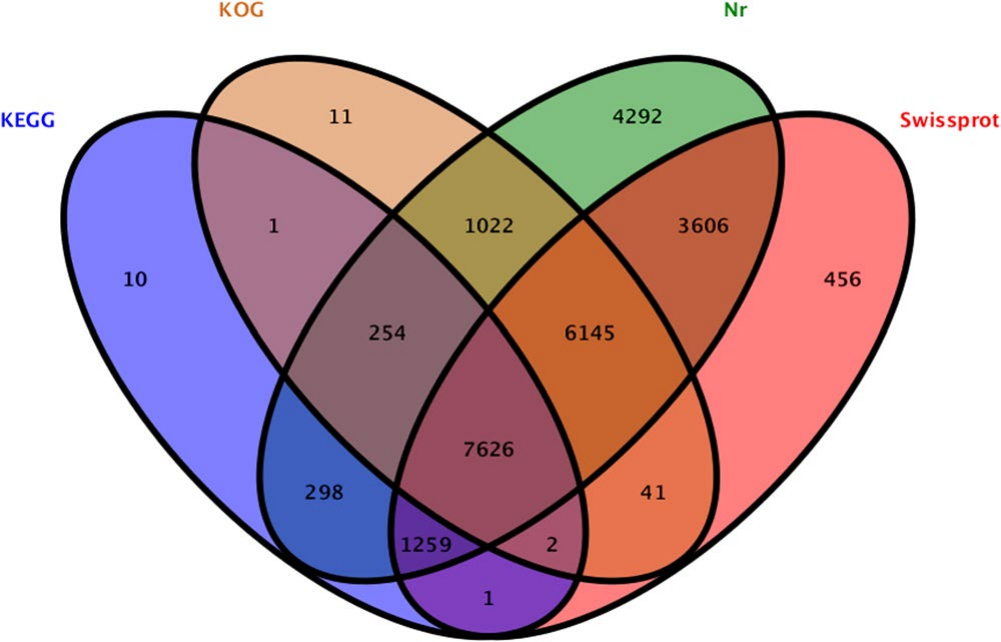
Venn diagram of annotated unigenes of *A*. *orientale*.

**Figure 3:**
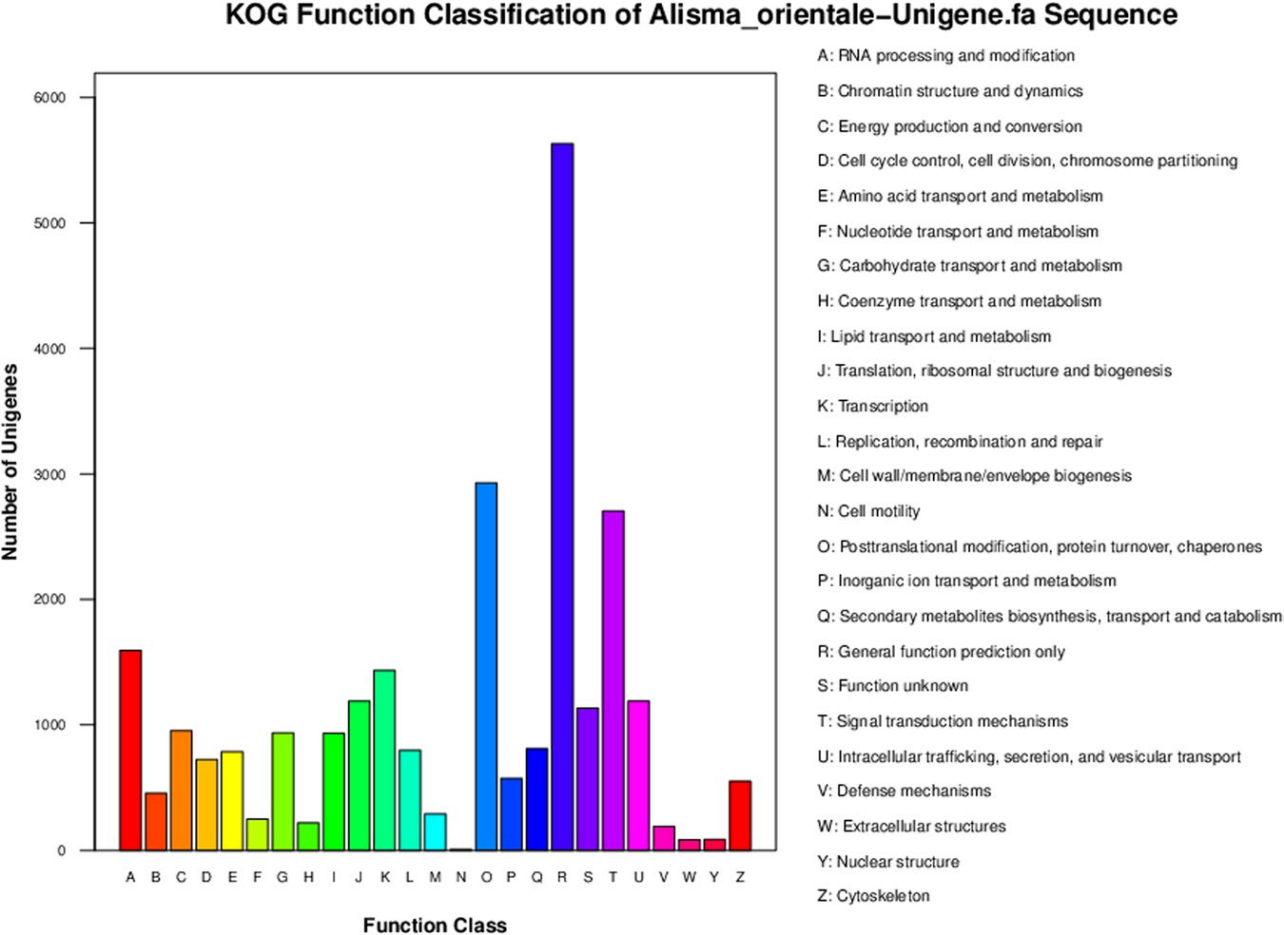
KOG function classifications of the *A*. *orientale* unigenes.

**Figure 4:**
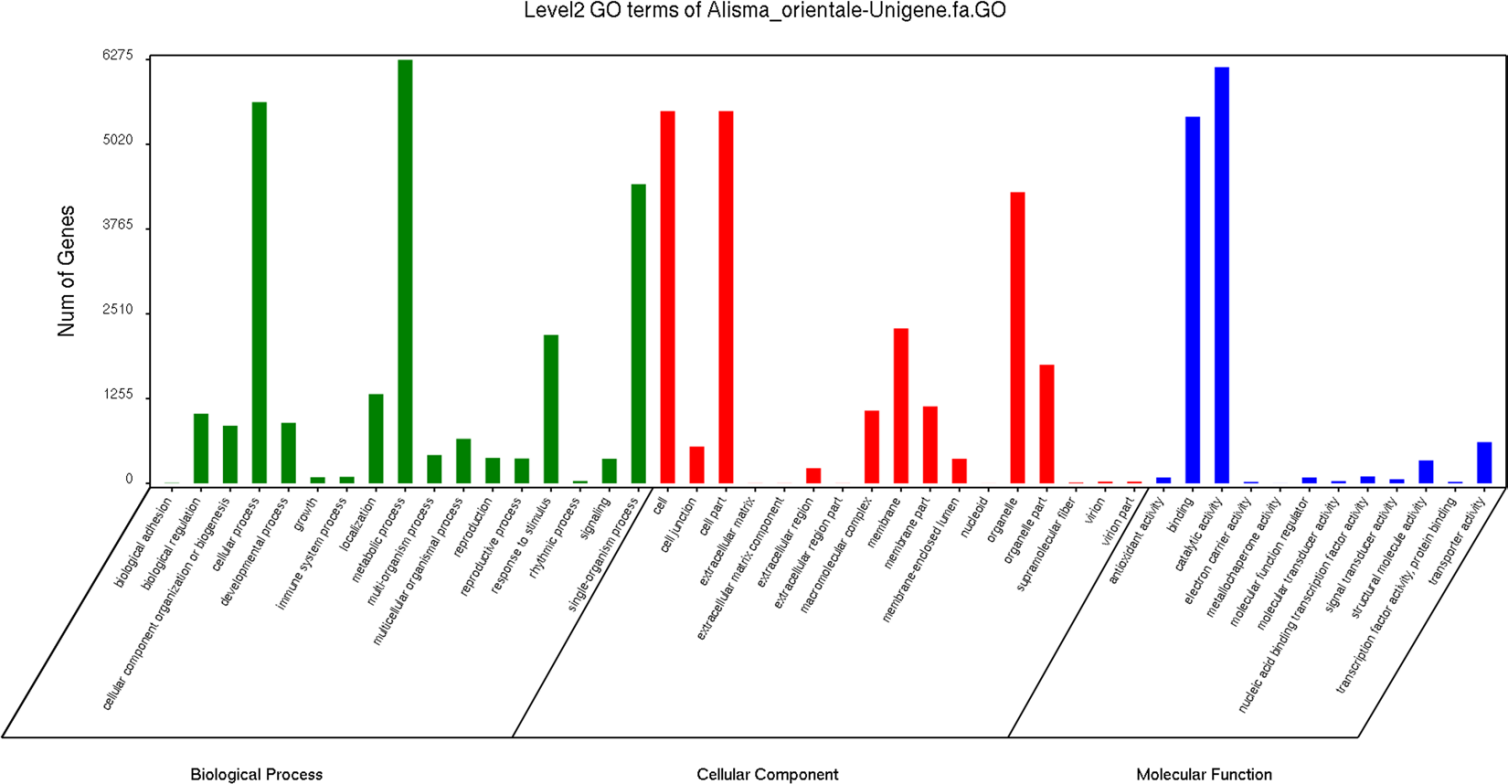
Level2 GO terms of the *A*. *orientale* unigenes.

A total of 25,024 unigenes were annotated against five public databases, non-redundant protein (Nr), Swiss-Prot protein (SwissProt), Gene Ontology (GO), Eukaryotic Orthologous Groups (KOG), and Kyoto Encyclopedia of Genes and Genomes (KEGG) (Fig. 2). A total of 15,102 unigenes were annotated and further functionally classified by the KOG databases. “General function prediction only (5,629)” was the largest proportion of the function classifications, followed by “posttranslational modification, protein turnover, chaperones (2,928)” and “signal transduction mechanisms (2,705)” (Fig. 3, Table S3). A total of 12,320 unigenes were divided into three GO categories and 46 functional classes after annotation by GO analysis. The metabolic process category (6,271) was the largest proportion of the GO annotations, followed by catalytic activity (6,163) and cellular processes (5,644) (Fig. 4, Table S4). These annotations will help further molecular studies of *A*. *orientale*.

### SSR Screening and analysis

**Figure 5:**
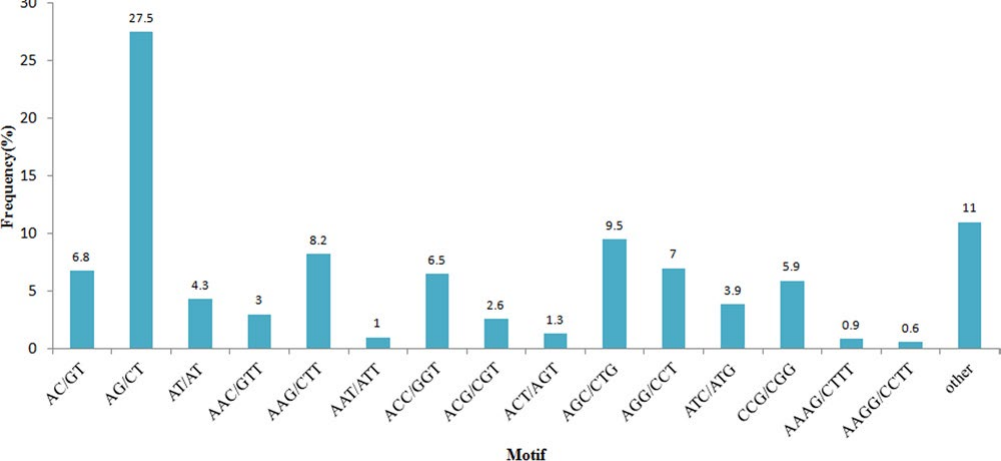
Frequencies of the SSRs identified in *A*. *orientale* unigenes.

To detect SSRs in *A*. *orientale*, a total of 41,685 sequences and 46,562,041 bp data were examined. There were 3,411 identified SSRs in 2,871 sequences, among which 423 sequences had more than one SSR, and 173 SSRs presented in compound formation. All SSRs were classified by their repeat unit sizes. The results of the repeat type SSR analysis showed that the length of the various SSR repeat types greatly differed (Table 1). The tri-nucleotide repeat type composed the largest portion (48.90%) of total SSRs, followed by di-nucleotides (38.82%) and tetra-nucleotides (6.71%). The penta-nucleotides and hexa-nucleotides had a fraction of less than 5%. The most frequent type of di-nucleotide repeats was AG/CT, which represented a large proportion (27.5%) of the di-nucleotides. Among all the tri-nucleotide motif types, AGC/CTG repeats accounted for the largest proportion of all SSR types, followed by AAG/CTT (8.2%), ACC/GGT (6.5%), and CCG/CGG (5.9%). The SSR types and frequencies of *A*. *orientale* are shown in Fig. 5. We have performed tissue-specific loci analysis in SSRs sequences. The result showed that, among the 2,871 SSRs sequences, 22 SSRs sequences were detected only in RT, six SSRs sequences were detected only in IN, which may be used as molecular markers of different tissues.

**Table 1:** Numbers of the SSR types in the transcriptome of *A*. *orientale*.

Number of repeat unit	Di-	Tri-	Tetra-	Penta-	Hexa
4	0	0	184	50	85
5	0	1095	31	4	37
6	509	346	10	1	2
7	284	151	2	0	3
8	193	36	1	0	2
9	139	22	0	0	1
10	88	9	0	0	1
11	49	3	0	1	0
12	14	1	0	0	0
13	2	0	0	0	0
14	10	2	0	0	0
>=15	36	3	1	0	3
Total	1324	1668	229	56	134
Percentage	38.82%	48.90%	6.71%	1.64%	3.93%

### Identification of DEGs

**Figure 6:**
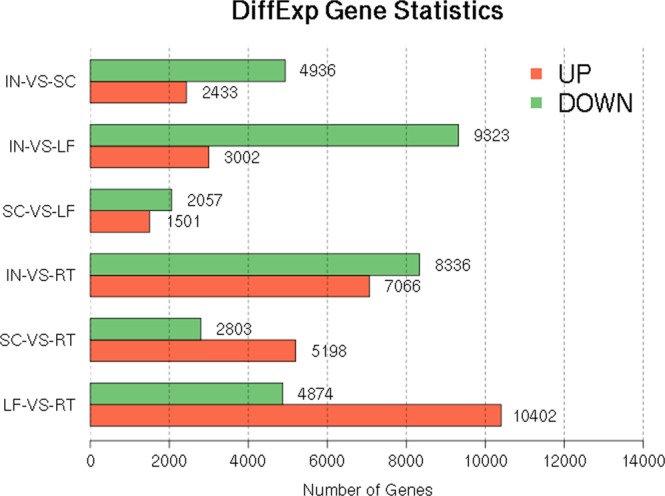
Annotated DEG numbers among various tissues of *A*. *orientale*.

When compared to each other (LF-VS-RT, SC-VS-RT, IN-VS-RT, SC-VS-LF, IN-VS-LF, IN-VS-SC), the distributions of differentially expressed genes were different in the four tissues. Among the six comparisons, SC-VS-LF accounted for the least number of DEGs (3,558), whereas 15,276 and 8,001 were detected in LF-VS-RT and SC-VS-RT, respectively, In addition, 15,402, 12,325 and 7,369 DEGs were detected in IN-VS-RT, IN-VS-LF and IN-VS-SC, respectively. These results indicated that the SC were more similar to young LF than to the others tissues and that the IN were different from the others tissues. The DEG statistical analysis is shown in Fig. 6. Compared with the genes in the IN, 3,002, 7,066 and 2,433 genes were upregulated, and 9,323, 8,336 and 4,936 genes were downregulated in the LF, RT and SC, respectively. The DEG expression analysis showed that IN was significantly different from RT, LF and SC, and that there was a similar pattern between SC and LF.

### Functional classification of the DEGs

To understand the functions of the DEGs, the GO and KEGG databases were employed to annotate the DEGs. GO enrichment analysis of the DEGs in pairs were performed, and in addition, to further clarify the difference between IN and the other three tissues (root, leaf and scape), GO enrichment analysis of the DEGs between the IN and the other three tissues (root, leaf and scape: RLS) were also performed. The numbers of DEGs annotated in IN-VS-RT (Fig. S5, Table S6) and IN-VS-LF (Fig. S7, Table S8) were higher than those in IN-VS-SC (Fig. S9, Table S10). The top three most frequent GO terms assigned to the biological process category were “cellular process”, “metabolic process,” and “single-organism process”, and the top three most frequent GO terms of cellular component GO category were “cell”, “cell part”, and “organelle”, The number of genes involved in “binding” and “catalytic activity” was greater than that involved in other GO terms in the molecular function category.

Among the 651 tissue-specific unigenes in RT, there were seven unigenes that were related to development, such as single-organism developmental process (Unigene0029170, Unigene0037640, Unigene0030866, Unigene0025820, Unigene0029058), developmental process involved in reproduction (Unigene0026217), regulation of meristem development (Unigene0001186); among the 111 tissue-specific unigenes in IN, there were four unigenes that were related to multicellular organism development (Unigene0017046, Unigene0004934, Unigene0023331, Unigene0013750); among the 27 tissue-specific unigenes in LF, there were five unigenes that were related to metabolic process (Unigene0035480, Unigene0007410, Unigene0006949, Unigene0017526, Unigene0036445); among the 27 tissue-specific unigenes in LF, there was one unigenes that were related to metabolic process (Unigene0040067) (Table S6).

An enriched KEGG pathway analysis of the DEGs among different the tissues of *A*. *orientale* was also performed. The DEGs were assigned to 341, 126, 124, and 122 pathways in IN-VS-RLS (Table S11), IN-VS-RT (Table S12), IN-VS-LF (Table S13) and IN-VS-SC, respectively (Table S14). The top 20 KEGG pathways in the four tissues are shown in Table 2. Interestingly, “spliceosome” pathways accounted for a small portion of the DEGs in IN-VS-RT but a large proportion in IN-VS-LF and IN-VS-SC. “Plant-pathogen interaction” accounted for a large portion of the DEGs in IN-VS-RT but a small proportion in IN-VS-LF and IN-VS-SC. In addition, “phenylpropanoid biosynthesis” had larger proportions of DEGs in both IN-VS-RT and IN-VS-SC than in IN-VS-LF, and accounted for the largest portion of DEGs in IN-VS-RLS (Fig. S15).

**Table 2:** Enriched KEGG pathway analysis of the DEGs between IN and the other three tissues.

Pathway	IN-VS-RT	Percent (%)	IN-VS-LF	Percent (%)	IN-VS-SC	Percent (%)
Ribosome	70	3.66	94	5.19	60	5.45
Carbon metabolism	115	6.01	110	6.07	33	3.00
Biosynthesis of amino acids	91	4.75	72	3.98	31	2.82
Plant hormone signal transduction	143	7.47	102	5.63	76	6.91
Protein processing in endoplasmic reticulum	59	3.08	78	4.31	34	3.09
Spliceosome	40	2.09	116	6.41	70	6.36
Starch and sucrose metabolism	115	6.01	60	3.31	49	4.45
Plant-pathogen interaction	118	6.16	49	2.71	47	4.27
Phenylpropanoid biosynthesis	149	7.78	72	3.98	75	6.82
Purine metabolism	53	2.77	73	4.03	53	4.82
Endocytosis	63	3.29	35	1.93	23	2.09
Oxidative phosphorylation	33	1.72	35	1.93	20	1.82
RNA transport	48	2.51	90	4.97	52	4.73
Glycolysis -VS- Gluconeogenesis	60	3.13	55	3.04	17	1.55
Ubiquitin mediated proteolysis	31	1.62	60	3.31	27	2.45
Pyrimidine metabolism	51	2.66	62	3.42	51	4.64
Amino sugar and nucleotide sugar metabolism	39	2.04	58	3.20	35	3.18
RNA degradation	39	2.04	25	1.38	18	1.64
mRNA surveillance pathway	21	1.10	62	3.42	30	2.73
Cysteine and methionine metabolism	40	2.09	73	4.03	54	4.91

### DEGs validation by qRT-PCR

**Figure 7:**
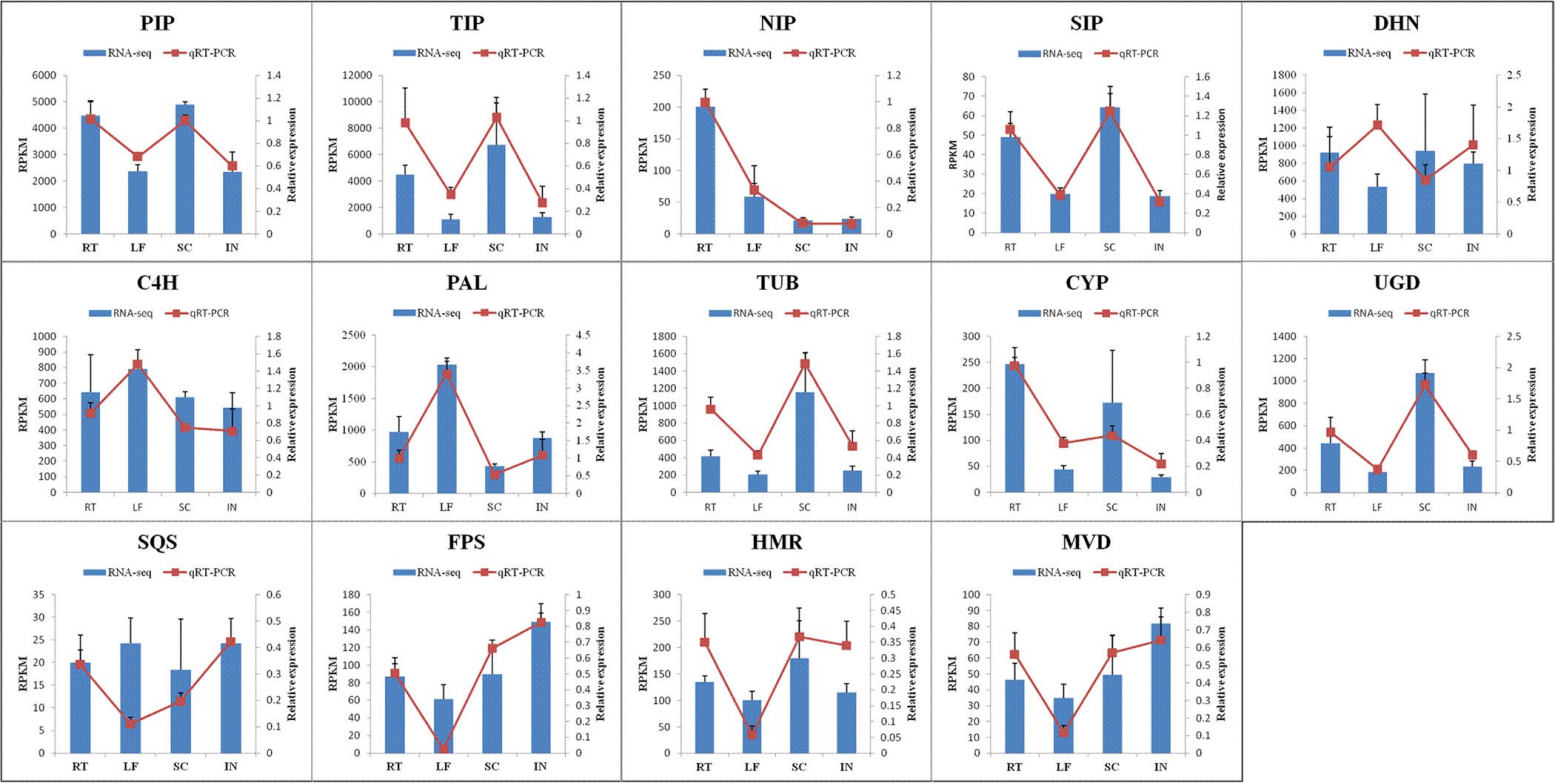
qRT-PCR validation of the DEGs involved in the biosynthesis of alisol and its derivatives. PIP: aquaporin PIP1-3, TIP: aquaporin TIP2-1, NIP: aquaporin NIP5-1, SIP: aquaporin SIP1-1, DHN: dehydrin, C4H: cinnamate 4-hydroxylase, PAL: phenylalanine ammonia-lyase, TUB: beta-tubulin 4, CYP: cytochrome P450 CYP73A100-like, UGD: UDP-glucose 6-dehydrogenase, SQS: farnesyl-diphosphate farnesyltransferase, FPS: farnesyl pyrophosphate synthase, HMR: 3-hydroxy-3-methylglutaryl-CoA reductase, and MVD: diphosphomevalonate decarboxylase.

In the present study, fourteen unigenes were chosen for qRT-PCR validation. These unigenes were annotated to aquaporin PIP1-3 (PIP), aquaporin TIP2-1 (TIP), probable aquaporin NIP5-1 (NIP), aquaporin SIP1-1 (SIP), dehydrin (DHN), cinnamate 4-hydroxylase (C4H), phenylalanine ammonia-lyase (PAL), beta-tubulin 4 (TUB), cytochrome P450 CYP73A100-like (CYP), UDP-glucose 6-dehydrogenase(UGD), farnesyl-diphosphate farnesyltransferase (SQS), farnesyl pyrophosphate synthase (FPS), 3-hydroxy-3-methylglutaryl-CoA reductase (HMR), and diphosphomevalonate decarboxylase (MVD). Most of the data from the qRT-PCR analysis were consistent with the transcriptome data (Fig. 7), except for the data on DHN and SQS. The expression levels of aquaporin PIP, TIP, SIP and CYP were higher in the RT and SC than in the LF and IN; the expression level of aquaporin NIP was higher in the RT than in the other tissues; the expression level of PAL was the highest in the LF; the expression levels of TUB and UGD were the highest in the SC; and there was no significant difference in the expression levels of C4H, DHN and SQS among the different tissues. The expression levels of the other three genes involved in the biosynthesis of alisol and its derivatives, TPS and MVD, were higher significantly in the IN than in the SC, RT and LF, and the expression level of HMR was the lowest in the LF and the highest in the SC. These results suggested that FPS and MVD could be the critical candidate genes involved in the biosynthesis of alisol and its derivatives.

### Contents of alisol and its derivatives

**Figure 8:**
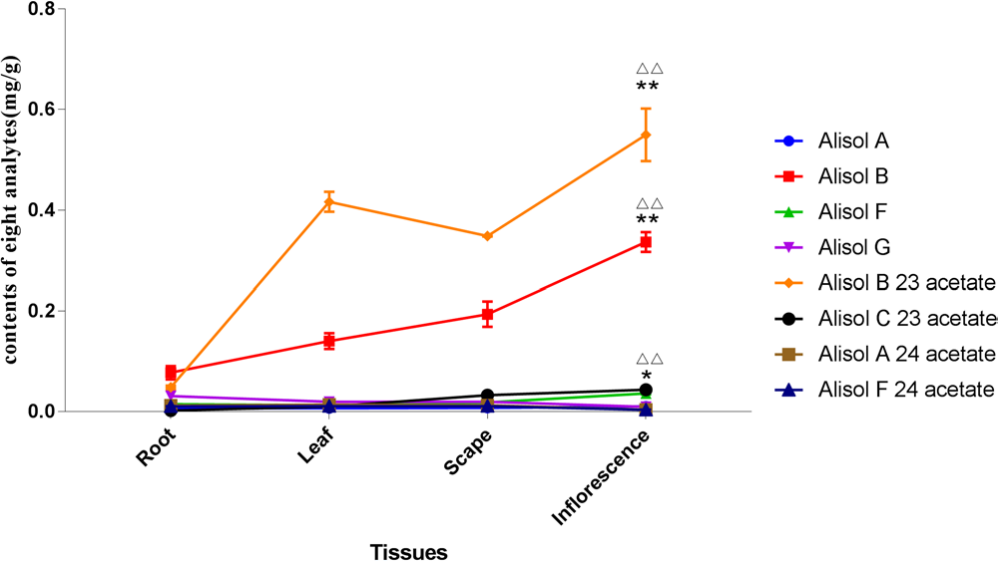
Contents of the eight triterpenoids in *A*. *orientale*. Inflorescence compared to root, ^ΔΔ^*p* < 0.01, inflorescence compared to leaf, ^*^p < 0.05, ^**^p < 0.01.

The analytical method developed was used to simultaneously determine the contents of eight components from four tissue samples of *A*. *orientale*. The retention time, linear regression data, and linear range of alisol A, alisol A 24-acetate, alisol B, alisol B 23-acetate, alisol C 23-acetate, alisol F, alisol F 24-acetate, and alisol G were listed in Table 3. The relative standard deviation (RSD) values of precision, stability and repeatability of the target components were 1.20~3.91%, 1.35~3.98% and 2.26~4.11%, respectively. The recovery rate of the eight reference substances were 97.15% ~104.20% (RSD ≤ 4.18%). In short, the method had good linearity, precision, repeatability, stability, and accuracy. These results indicated that the developed method is suitable for the quantitative determination of the eight components in the *A*. *orientale* samples. The contents of the components are shown in Fig. 8. The results indicated that all eight analytes could be detected in the four tissues. Alisol B 23-acetate and alisol B were the most abundant compounds, and the contents of alisol B 23-acetate were higher than those of alisol B in the IN, SC and LF, but lower than those of alisol B in the RT. The contents of alisol B 23-acetate and alisol B were much higher in the IN and SC than in the RT and LF tissues, and the contents of Alisol C 23-acetate were obviously higher in the IN than in RT and LF tissues. Between the IN and SC, the concentrations of the eight types of alisol and its derivative showed no significant changes.

**Table 3:** Retention time, linear regression data, linear range, and contents of eight triterpenoids from UPLC-QQQ-MS quantification.

Analytes	t_R_(min)	Calibration Curve	Linear Range (μg/mL)	r^2^
Alisol F	4.29	y = 0.2808x–0.0002	0.0100–10.0	0.9975
Alisol C 23- acetate	4.31	y = 1.0021x–0.0004	0.0104–10.4	0.9989
Alisol F 24- acetate	4.8	y = 0.408x–0.0021	0.0106–10.6	0.9998
Alisol A	5.09	y = 0.3685x–0.0011	0.0098–9.8	0.9997
Alisol A 24- acetate	5.9	y = 0.1125x–0.0006	0.0102–10.2	0.9975
Alisol G	6.62	y = 1.1373x–0.0037	0.0100–10.0	0.9965
Alisol B	6.87	y = 0.5238x–0.0098	0.0104–10.4	0.9993
Alisol B 23 acetate	7.85	y = 0.0646x–0.0012	0.0098–9.8	0.9993

## Discussion

*A*. *orientale* belongs to the genus *Alisma* genus, as well as to *Alismataceae*, Alismatineae, Helobiae, and Monocotyledoneae of the Angiospermae. There are approximatelyabout 11 species of *Alisma* in the temperate and subtropical regions; there are six species (one endemic) in China, namely, *Alisma canaliculatum*, *Alisma gramineum*, *Alisma lanceolatum*, *Alisma nanum*, *Alisma orientale* (Samuel.) Juz, and *Alisma plantago-aquatica* Linn. The Royal Botanic Gardens Kew DNA C-values database (http://data.kew.org/)^46^ query results showed that the mean C-value of *Alism*a is 15.05. Based on this calculation^47^, the genome size of *Alisma* was estimated to be 14.72Gb^46,47^. To date, only 39 nucleotide sequences were available for *A*. *orientale* in NCBI GenBank. In the present study, transcriptome sequencing of mRNA samples from the RT, LF, SC and IN of *A*. *orientale* and de novo assembly were performed. A total of 372,971,128 high-quality reads and 41,685 unigenes were obtained. Approximately 60% of the unigenes (25,024 out of 41,685) were annotated by BLASTx against five public databases, and most of the remaining unigenes of *A*. *orientale* need to be functionally annotated in the future. To our knowledge, this is the first study on the transcriptome of *A*. *orientale*. These unigenes provide a better understanding of the molecular mechanism related to the medicinal features of *A*. *orientale*.

Because SSRs are highly polymorphic and adapt to different species, they have been widely used in plant genetic and breeding studies, such as genetic map construction^48,49^, population genetic research^50^, genetic diversity estimation^51^ and germplasm conservation^52^. There were no *Alisma* ESTs in the NCBI databases utill now. Further genetic analyses of *A*. *orientale* require *the deve*lopment of more species-specific SSR makers. Therefore, the 3,411 SSRs that were identified in this study will greatly increase the amount of the existing information on microsatellites in *A*. *orientale*, which will further help elucidate the important genetic traits of medicinal plants and help support the molecular marker-assisted breeding and conservation of medicinal plants. In the next step, we will use genetic SSR markers to assess the genetic diversity, population structure and genetic relationship of *A*. *orientale* and *A*. *plantago*.

In addition, the comparison of the DEGs showed that the difference between the scapes and leaves of *A*.*orientale* was the smallest, indicating that the development of scapes and leaves is very similar. The difference between the IN and RT was the largest, especially in the pathways of phenylpropanoid biosynthesis, starch and sucrose metabolism, plant hormone signal transduction, and plant-pathogen interaction, the expression levels of the related DEGs were lower in the IN than in the RT. The difference between RT and LF was also very large, mainly in the pathways of phenylpropanoid biosynthesis, plant hormone signal transduction, carbon metabolism, plant-pathogen interaction, starch and sucrose metabolism and biosynthesis of amino acids. In general, unlike in the other tissues, the number of upregulated genes in the LF was lower than the number of downregulated genes; the number of upregulated genes in the IN was greater than the number of downregulated genes.

*A*. *orientale* is an aquatic monocotyledonous plant, and water is very important to it. Aquaporins are a superfamily of integral membrane proteins, which play an important role in the transportation of water between cells^53^. To date, little is known about the aquaporin genes in *A*. *orientale*. In this study, the aquaporin unigenes were divided into four categories including PIP, TIP, NIP, and SIP. To adapt to the aquatic environment, the RT and SC of *A*. *orientale* have a number of vessels and aerenchyma. The differences in the tissue microstructures showed that compared with the other tissues, the LF had more chlorophyll, the SC had more vascular bundles, and the IN contained more starch granules and protein, which were consistent with the RNA-seq data. The expression levels of aquaporin PIP, TIP, NIP, and SIP were higher in the RT than in the LF and IN. The pathway enrichment analysis indicated that DEGs related to “photosynthesis”, “carbon fixation in photosynthetic organisms”, “porphyrin and chlorophyll metabolism” and “starch and sucrose metabolism” were more numerous and more highly expressed in LF, SC and IN than in the RT.

*A*. *orientale* is a genuine medicinal material in Fujian Province, China. The genetic characteristics of *A*. *orientale* and the molecular basis for the synthesis of its active ingredients are still not clear. Protostane triterpenoids are the characteristic components of *A*. *orientale*^2^. Among these protostane triterpenoids, alisol B 23-acetate and alisol B were the major components in the four tissues, at amounts of 0.068~0.350 mg/g and 0.046~0.587 mg/g, respectively. These results were the same as those in the tuber of *A*. *orientale*^54^ and were in accordance with the results from a previous report^55^. When the four tissues were compared, there were obvious differences between the RT and the other tissues; alisol B was higher than alisol B 23-acetate in the RT. For the other components, alisol A, alisol A 24-acetate, alisol F, alisol F 24-acetate and alisol G, were not significantly different in quantity among the four tissues. The alisol B 23-acetate and alisol B contents were higher in the IN than in the other three tissues. Moreover, only in IN, the content of alisol B 23-acetate was above 0.5 mg/g, which is the limit standard of alisol B 23-acetate content in the Pharmacopoeia of the People’s Republic of China. These results were consistent with transcriptome sequencing data, and RT-PCR validated that FPS and MVD were the key candidate genes involved in the biosynthesis of alisol and its derivatives. These results suggested that the IN of *A*. *orientale* could be used as a new edible and medical substance. Moreover, to facilitate the comprehensive development and utilization of *A*. *orientale*, the leaves of *A*. *orientale* can also be used to extract and refine the active ingredients, such as alisol B 23-acetate, alisol B and other active triterpenoids.

## Materials and Methods

### Plant material collection

The plant samples of *A*. *orientale* were obtained from Jian’ou County, Nanping City, Fujian Province in July 2016. The height of the collected plants height was approximately 45 cm, and the inflorescences had not yet flowered, and the plants were identified as *Alisma orientale* (Sam.) Juzep by Zhuqing Lv at Station for Popularizing Agricultural Technique of Nanping City. Roots (RT), leaves (LF), scapes (SC) and inflorescences (IN) from three biological replicates of *A*. *orientale* were harvested, immediately frozen in liquid nitrogen, and then stored in ultra cold freezers at −80 °C, respectively. The RT, LF, SC and IN from another three biological replicates of fresh *A*. *orientale* were rapidly put into formalin-acetic acid alcohol (FAA) fixative solution. Each biological replicate used three individual plants.

### Microscopic structure analysis

Fresh samples of RT, LF, SC and IN were quickly sliced horizontally and vertically and placed into FAA fixative solution for 24 h, Then, the samples were dehydrated by a gradient series of ethanol and embedded into paraffin blocks for microscopic structure observation. The samples were cut with a LEICA RM2016 microtome (LEICA, Solms, German), deparaffinized, hydrated in water(xylene, 20 min, twice,100% alcohol, 5 min, twice, 75% alcohol, 5 min, and rinsed in water), and stained with I-KI for a starch grain analysis, or fast green for a cutinized or cellulosic tissue analysis. Each slice was analysed using a Nikon Eclipse CI microscope and then photographed using the Nikon DS-U3 imaging system (Nikon, Tokyo, Japan).

### RNA isolation and sequencing

According to the manufacturer’s instructions, the RNAsimple Total RNA Kit (Tiangen, Beijing, China) was used to extract total RNA from the RT, LF, SC and IN of three batches of *A*. *orientale*. The quality and quantity of RNA were examined by spectrophotometric analysis (BioDrop spectrophotometer, BioDrop Technologies, Cambridge, UK) and agarose gel electrophoresis. After total RNA was extracted, the mRNA was enriched with magnetic beads, cut into short fragments, and then reverse transcribed into first-strand cDNA. The second-strand cDNA were synthesized by RNase H, DNA polymerase I and dNTPs. Next, the cDNA fragments was purified and ligated to Illumina sequencing adapters. The ligation products were amplified by polymerase chain reaction, followed by high-throughput sequencing on the Illumina HiSeqTM 4000 platform (Gene Denovo Biotechnology Co. Ltd, Guangzhou, China).

### De Novo assembly and basic functional annotation

The original reads of the transcriptome sequences were filtered to acquire the high-quality reads, and then the low-quantity reads, including the sequencing adaptor, reads with the “N” percentage exceeding 10% or above 40% bases with a Q-value ≤ 20, were removed. Transcriptome de novo assembly was performed using the common Trinity program^56^. The basic functional annotation includes annotation of protein function, pathway, Eukaryotic Orthologous Groups (KOG), and Gene Ontology (GO)^57^. The BLASTx program (http://www.ncbi.nlm.nih.gov/BLAST/) was used to annotate the protein function against the non-redundant protein (Nr) database (http://www.ncbi.nlm.nih.gov) and Swiss-Prot protein database (http://www.expasy.ch/sprot) with an E-value of 1E-5. The Kyoto Encyclopedia of Genes and Genomes (KEGG) database (http://www.genome.jp/kegg) was employed for pathway annotation, and the COG/KOG database (http://www.ncbi.nlm.nih.gov/COG) was employed for COG/KOG annotation. The Blast2GO software was employed for GO annotation^58^. WEGO software was employed for the functional classification annotation of the unigenes^59,60^. Venn Diagrams were plotted using the OmicShare tools, a free online platform for data analysis (www.omicshare.com/tools).

### Simple sequence repeat prediction

MIcroSAtellite (MISA, http://pgrc.ipk-gatersleben.de/misa/) was used to predict simple sequence repeats (SSRs) in the whole transcriptome^61^. The parameters were as follows: definition (unit_size, min_repeats), 2-6 3-5 4-4 5-4 6-4, and interruptions (max_difference_between_2_SSRs), 100. Potential SSR markers ranging from one to six nucleotides in length were detected in all assembled unigenes.

### Differentially expressed genes identified

The abundances of all unigenes in the RT, LF, SC and IN of *A*. *orientale* were calculated and normalized to reads per kb per million reads (RPKM)^62^. The edgeR package (http://www.r-project.org/) was employed to identify the differentially expressed genes (DEGs) among the RT, LF, SC and IN transcriptome libraries. A fold change >2 and a false discovery rate (FDR) < 0.05 were used to identify significant DEGs. The DEGs were then used for enrichment analysis of the KEGG pathways and GO functions.

### Analysis of qRT-PCR

Total RNA was extracted from approximately 100 mg frozen RT, LF, SC and IN of *A*. *orientale* with Trizol^TM^ reagent (Invitrogen, USA). The PrimeScript^TM^ RT reagent kit with gDNA Eraser (RR047A, TaKaRa, CHINA) was employed to reverse transcribes the RNA into cDNA. Ten high expression unigenes aquaporin PIP1-3 (Unigene0025824), aquaporin TIP2-1 (Unigene0026873), probable aquaporin NIP5-1 (Unigene0012384), aquaporin SIP1-1 (Unigene0030382), dehydrin (Unigene0032085), cinnamate 4-hydroxylase (Unigene0020543), phenylalanine ammonia-lyase (Unigene0032343), beta-tubulin 4 (Unigene0013978), cytochrome P450 CYP73A100-like (Unigene0014409), and UDP-glucose 6-dehydrogenase (Unigene0024355), and four candidate unigenes, farnesyl-diphosphate farnesyltransferase (Unigene0015281), farnesyl pyrophosphate synthase (Unigene0029957), 3-hydroxy-3-methylglutaryl-CoA reductase (Unigene0012787), and diphosphomevalonate decarboxylase (Unigene0020140) involved in the biosynthesis of alisol and its derivatives were selected for qRT-PCR analysis. The internal control gene (*β-*Actin) and the candidate unigene optimized primers were designed by Primer 3 software (http://bioinfo.ut.ee/primer3/) (Table S16). The cDNA samples from the different tissues of *A*. *orientale* were analysed by qRT-PCR using SYBR® Premix Ex Taq II (Takara Co. Ltd) in an ABI 7500 Real Time Detection System (Applied Biosystems, USA). The PCR cycle parameters were as follows: 95 °C for 30 s, 40 cycles of 95 °C for 5 s and 58 °C for 30 s. The qRT-PCR was performed using three biological and three technical replicates. All reactions were repeated in triplicate.

### Determination of the contents of alisol and its derivatives

Eight reference substances alisol A, alisol A 24-acetate, alisol B, alisol B 23-acetate, alisol C 23-acetate, alisol F, alisol F 24-acetate, and alisol G, were purchased from MANSITE Biotechnology Co. Ltd, Chendu, China. Quantitative analysis of eight triterpenoids was performed on the Waters TQ TQS system of ultra high-performance liquid chromatography configured with a triple quadruple mass spectrometer (UPLC-QQQ-MS, Waters, UK), according to previous research^54^. First, each reference substance was separately dissolved in acetonitrile to approximately 0.5 mg/mL; then, the stock solution of each reference substance was diluted to obtain a series of working solutions to establish a calibration curve. Formic acid (0.1%) solution (A) and acetonitrile (B) constituted the mobile phase, and the elution gradient programme was as follows: 30~55% B at 1.0~1.5 min, 55~75% B at 1.5~5.5 min, 75~90% B at 5.5~7.5 min, 90~90% B at 7.5~8.5 min, 90~30% B at 8.5~8.6 min, 30~30% B at 8.6~10 min. The flow rate was 0.25 mL/min, the injection volume was 2.0 μL, and the nebulizer gas was 800 L/h at 500 °C. Three biological replicates of the four tissues from *A*. *orientale* were separately ground into fine powder and thoroughly mixed. Each sample was precisely weighted (0.5 g) and extracted with 25 mL acetonitrile for 30 min in an ultrasonic bath. The extraction solution was filtered through a 0.22 μm syringe filter membrane. The filtrate was then diluted 20 times, mixed with glycyrrhetinic acid (5.1 mg/L) internal standard solution, and filtered through a 0.22 μm syringe filter membrane for analysis.

### Accession codes

The transcriptome data of *A. orientale* that were analysed during this study are available in the NCBI SRA repository, https://trace.ncbi.nlm.nih.gov/Traces/sra_sub/sub.cgi?acc=SRP124598.

## Supplementary information

Supplementary Figures and tables
