# Supplementary material for: Comparative transcriptome and metabolite profiling of four tissues from Alisma orientale (Sam.) Juzep reveals its inflorescence developmental and medicinal characteristics
Source: Sci Rep. 2019 Aug 23;9:12310. doi: 10.1038/s41598-019-48806-w (PMC6707231; doi:10.1038/s41598-019-48806-w)
Supplement: Supplementary file 1 — Supplementary Figures and tables [file 41598_2019_48806_MOESM1_ESM.pdf]

Comparative transcriptome and metabolite profiling of four tissues from *Alisma orientale* (Sam.) Juzep reveals its inflorescence developmental and medicinal characteristics

Wenjin Lin[1*], Fengling Sun[1], Yamin Zhang[1], Xiaomei Xu[1], Xuehua Lu[1], Lisha Li[1], Rongqing Xu[1*]

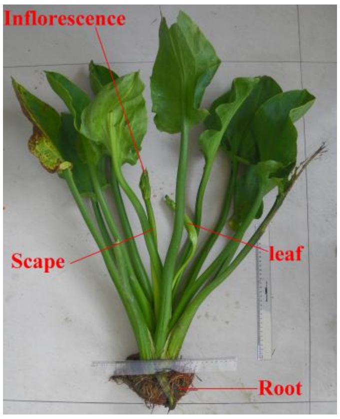

Figure S1| Root, leaf, scape and inflorescence of *A. orientale*

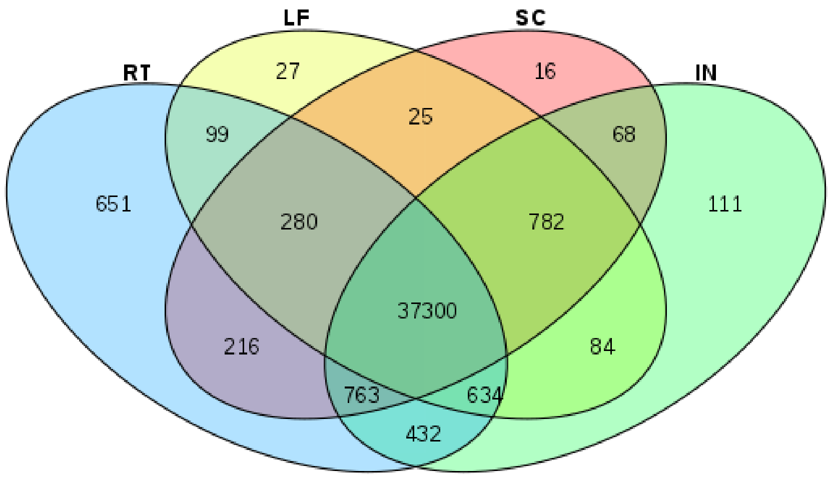

Figure S2| Venn diagram of tissue-specific loci unigenes. RT:Root, LF:leaf, SC:scape, IN:inflorescence

Table S3 KOG class annotation of *A. orientale* unigenes.

Code	Functional-Categories	Gene-Number
A	RNA processing and modification	1594
B	Chromatin structure and dynamics	454
C	Energy production and conversion	954
D	Cell cycle control, cell division, chromosome partitioning	724
E	Amino acid transport and metabolism	785
F	Nucleotide transport and metabolism	249
G	Carbohydrate transport and metabolism	936
H	Coenzyme transport and metabolism	220
I	Lipid transport and metabolism	933
J	Translation, ribosomal structure and biogenesis	1188
K	Transcription	1434
L	Replication, recombination and repair	797
M	Cell wall/membrane/envelope biogenesis	290
N	Cell motility	8
O	Posttranslational modification, protein turnover, chaperones	2928
P	Inorganic ion transport and metabolism	572
Q	Secondary metabolites biosynthesis, transport and catabolism	810
R	General function prediction only	5629
S	Function unknown	1134
T	Signal transduction mechanisms	2705
U	Intracellular trafficking, secretion, and vesicular transport	1190
V	Defense mechanisms	189
W	Extracellular structures	84
Y	Nuclear structure	88
Z	Cytoskeleton	551

Table S4 Level2 GO terms of the *A. orientale* unigenes.

Ontology	Class	number_of_Alisma_orientale Unigene.fa.GO
**Biological Process**	reproduction	377
**Biological Process**	immune system process	96
**Biological Process**	metabolic process	6271
**Biological Process**	cellular process	5644
**Biological Process**	reproductive process	368
**Biological Process**	biological adhesion	5
**Biological Process**	signaling	364
**Biological Process**	multicellular organismal process	659
**Biological Process**	developmental process	896
**Biological Process**	growth	91
**Biological Process**	single-organism process	4429
**Biological Process**	rhythmic process	34
**Biological Process**	response to stimulus	2198
**Biological Process**	localization	1322
**Biological Process**	multi-organism process	419
**Biological Process**	biological regulation	1032
**Biological Process**	cellular component organization or	853
**Molecular Function**	transcription factor activity, protein	22
**Molecular Function**	nucleic acid binding transcription	103
**Molecular Function**	catalytic activity	6163
**Molecular Function**	signal transducer activity	60
**Molecular Function**	structural molecule activity	340
**Molecular Function**	transporter activity	610
**Molecular Function**	binding	5428
**Molecular Function**	electron carrier activity	21
**Molecular Function**	antioxidant activity	86
**Molecular Function**	metallochaperone activity	1
**Molecular Function**	molecular transducer activity	33
**Molecular Function**	molecular function regulator	86
**Cellular Component**	extracellular region	224
**Cellular Component**	cell	5512
**Cellular Component**	nucleoid	1
**Cellular Component**	membrane	2293
**Cellular Component**	virion	26
**Cellular Component**	cell junction	545
**Cellular Component**	extracellular matrix	2
**Cellular Component**	membrane-enclosed lumen	364
**Cellular Component**	macromolecular complex	1076
**Cellular Component**	organelle	4311
**Cellular Component**	extracellular matrix component	2
**Cellular Component**	extracellular region part	2
**Cellular Component**	organelle part	1755
**Cellular Component**	virion part	26
**Cellular Component**	membrane part	1139
**Cellular Component**	cell part	5512
**Cellular Component**	supramolecular fiber	11

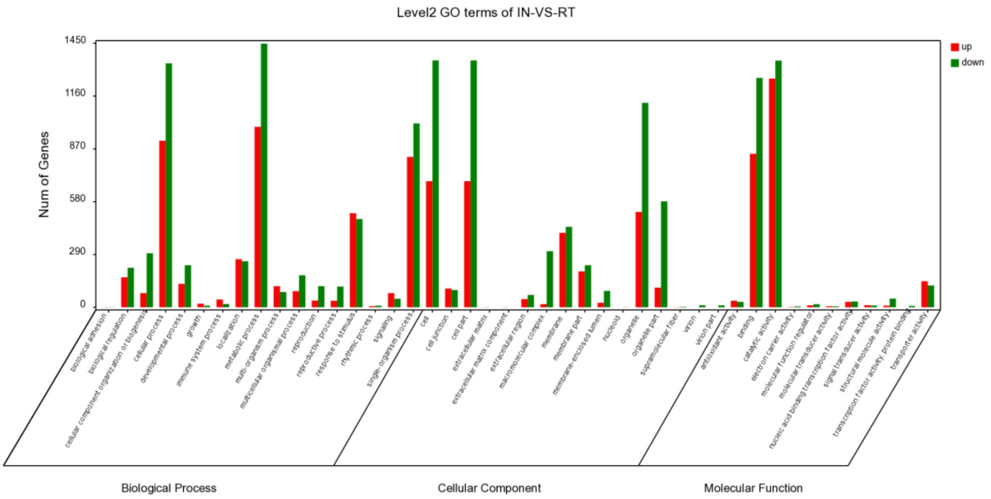

Figure S5 | GO enrichment analysis between the IN and RT of *A. orientale*.

Table S6 Level2 GO terms of *A. orientale* unigenes between IN and RT.

Ontology	Class	number_of_IN VS-RT_up	number_of_IN VS-RT_down
**Biological Process**	response to stimulus	517	485
**Biological Process**	immune system process	43	18
**Biological Process**	multi-organism process	116	84
**Biological Process**	cellular component organization or biogenesis	78	297
**Biological Process**	single-organism process	826	1010
**Biological Process**	rhythmic process	6	9
**Biological Process**	reproductive process	36	114
**Biological Process**	reproduction	37	116
**Biological Process**	multicellular organismal process	89	176
**Biological Process**	developmental process	129	231
**Biological Process**	biological adhesion	1	1
**Biological Process**	localization	264	253
**Biological Process**	cellular process	915	1340
**Biological Process**	growth	20	9
**Biological Process**	biological regulation	165	217
**Biological Process**	signaling	78	47
**Biological Process**	metabolic process	991	1448
**Molecular Function**	antioxidant activity	36	30
**Molecular Function**	catalytic activity	1256	1355
**Molecular Function**	nucleic acid binding transcription factor activity	30	32
**Molecular Function**	transporter activity	143	120
**Molecular Function**	transcription factor activity, protein binding	0	8
**Molecular Function**	signal transducer activity	12	10
**Molecular Function**	electron carrier activity	2	5
**Molecular Function**	molecular transducer activity	6	5
**Molecular Function**	molecular function regulator	11	17
**Molecular Function**	binding	843	1260
**Molecular Function**	structural molecule activity	9	48
**Cellular Component**	extracellular region	45	68
**Cellular Component**	organelle part	108	582
**Cellular Component**	organelle	524	1123
**Cellular Component**	nucleoid	0	1
**Cellular Component**	virion	0	11
**Cellular Component**	virion part	0	11
**Cellular Component**	extracellular matrix	1	0
**Cellular Component**	extracellular matrix component	1	0
**Cellular Component**	membrane part	197	231
**Cellular Component**	supramolecular fiber	1	3
**Cellular Component**	cell junction	103	95
**Cellular Component**	membrane	409	442
**Cellular Component**	membrane-enclosed lumen	25	90
**Cellular Component**	cell	693	1356
**Cellular Component**	cell part	693	1356
**Cellular Component**	macromolecular complex	17	308

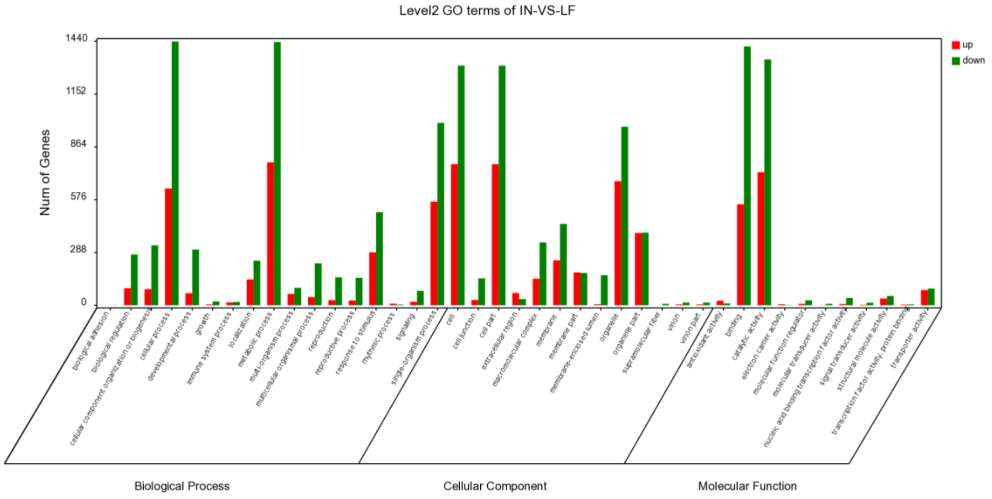

Figure S7 | GO enrichment analysis between the IN and LF of *A. orientale*.

Table S8 Level2 GO terms of *A. orientale* unigenes between IN and LF.

Ontology	Class	number_of_IN VS-LF_up	number_of_IN VS-LF_down
**Biological Process**	cellular component organization or biogenesis	88	327
**Biological Process**	reproduction	28	153
**Biological Process**	reproductive process	26	150
**Biological Process**	developmental process	66	304
**Biological Process**	cellular process	637	1439
**Biological Process**	multicellular organismal process	45	229
**Biological Process**	response to stimulus	289	508
**Biological Process**	multi-organism process	62	95
**Biological Process**	biological regulation	93	277
**Biological Process**	rhythmic process	9	4
**Biological Process**	immune system process	16	18
**Biological Process**	metabolic process	780	1436
**Biological Process**	single-organism process	565	995
**Biological Process**	biological adhesion	0	1
**Biological Process**	growth	5	21
**Biological Process**	signaling	19	79
**Biological Process**	localization	141	243
**Molecular Function**	binding	551	1412
**Molecular Function**	nucleic acid binding transcription factor activity	7	40
**Molecular Function**	antioxidant activity	25	10
**Molecular Function**	molecular function regulator	8	27
**Molecular Function**	transcription factor activity, protein binding	4	5
**Molecular Function**	electron carrier activity	6	2
**Molecular Function**	molecular transducer activity	1	8
**Molecular Function**	signal transducer activity	3	14
**Molecular Function**	catalytic activity	726	1341
**Molecular Function**	transporter activity	83	91
**Molecular Function**	structural molecule activity	37	51
**Cellular Component**	organelle part	394	396
**Cellular Component**	macromolecular complex	145	343
**Cellular Component**	cell	770	1307
**Cellular Component**	cell part	770	1307
**Cellular Component**	organelle	677	974
**Cellular Component**	membrane-enclosed lumen	6	164
**Cellular Component**	virion	5	15
**Cellular Component**	virion part	5	15
**Cellular Component**	extracellular region	67	34
**Cellular Component**	supramolecular fiber	0	8
**Cellular Component**	cell junction	29	147
**Cellular Component**	membrane part	179	176
**Cellular Component**	membrane	245	444

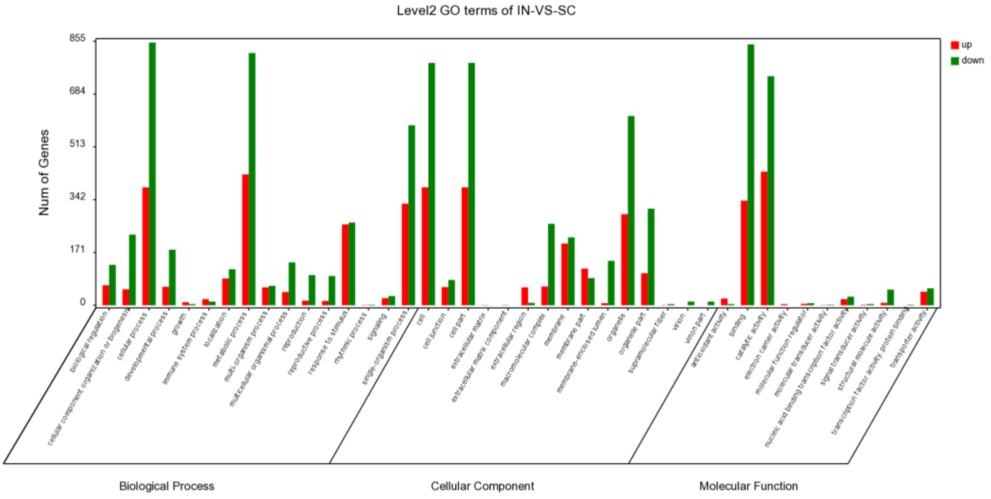

Figure S9 | GO enrichment analysis between the IN and SC of *A. orientale*.

Table S10 Level2 GO terms of *A. orientale* unigenes between IN and SC.

Ontology	Class	number_of_IN VS-SC_up	number_of_IN VS-SC_down
**Biological Process**	cellular component organization or biogenesis	52	229
**Biological Process**	developmental process	60	180
**Biological Process**	reproduction	15	98
**Biological Process**	multicellular organismal process	43	139
**Biological Process**	reproductive process	14	95
**Biological Process**	multi-organism process	58	63
**Biological Process**	response to stimulus	262	268
**Biological Process**	immune system process	20	12
**Biological Process**	cellular process	382	851
**Biological Process**	growth	10	4
**Biological Process**	single-organism process	329	583
**Biological Process**	biological regulation	65	131
**Biological Process**	rhythmic process	1	2
**Biological Process**	signaling	23	30
**Biological Process**	metabolic process	424	817
**Biological Process**	localization	87	117
**Molecular Function**	nucleic acid binding transcription factor activity	20	28
**Molecular Function**	binding	339	845
**Molecular Function**	antioxidant activity	22	4
**Molecular Function**	electron carrier activity	4	0
**Molecular Function**	structural molecule activity	8	51
**Molecular Function**	molecular function regulator	5	7
**Molecular Function**	transcription factor activity, protein binding	0	2
**Molecular Function**	molecular transducer activity	1	2
**Molecular Function**	signal transducer activity	2	4
**Molecular Function**	transporter activity	44	55
**Molecular Function**	catalytic activity	433	742
**Cellular Component**	membrane-enclosed lumen	7	144
**Cellular Component**	macromolecular complex	61	264
**Cellular Component**	organelle part	104	313
**Cellular Component**	extracellular region	58	8
**Cellular Component**	cell junction	59	82
**Cellular Component**	virion	0	12
**Cellular Component**	virion part	0	12
**Cellular Component**	supramolecular fiber	1	4
**Cellular Component**	cell	382	785
**Cellular Component**	cell part	382	785
**Cellular Component**	extracellular matrix	1	0
**Cellular Component**	extracellular matrix component	1	0
**Cellular Component**	organelle	295	613
**Cellular Component**	membrane part	119	88
**Cellular Component**	membrane	200	220

Table S11 Enriched KEGG pathway analysis of DEGs between IN and RLS.

KEGG_A_class	KEGG_B_class	Pathway	IN-VS-RLS (241)	All (5135)	Pvalue	Qvalue	Pathway ID
**Genetic Information Processing**	Replication and repair	Mismatch repair	16	48	1.99E-10	1.43E-08	ko03430
**Genetic Information Processing**	Replication and repair	Base excision repair	15	52	7.25E-09	2.61E-07	ko03410
**Genetic Information Processing**	Replication and repair	DNA replication	15	60	6.14E-08	1.47E-06	ko03030
**Metabolism**	Biosynthesis of other secondary metabolites	Phenylpropanoid biosynthesis	26	196	1.08E-06	1.94E-05	ko00940
**Genetic Information Processing**	Replication and repair	Homologous recombination	12	58	1.13E-05	1.63E-04	ko03440
**Genetic Information Processing**	Replication and repair	Non-homologous end-joining	5	12	0.000132	1.44E-03	ko03450
**Metabolism**	Lipid metabolism	Cutin, suberine and wax biosynthesis	7	26	0.00014	1.44E-03	ko00073
**Metabolism**	Nucleotide metabolism	Pyrimidine metabolism	16	129	0.000307	2.76E-03	ko00240
**Genetic Information Processing**	Replication and repair	Nucleotide excision repair	10	71	0.001586	1.27E-02	ko03420
**Metabolism**	Nucleotide metabolism	Purine metabolism	17	185	0.005703	4.11E-02	ko00230
**Genetic Information Processing**	Translation	Ribosome biogenesis in eukaryotes	11	108	0.011882	7.78E-02	ko03008
**Metabolism**	Amino acid metabolism	Lysine degradation	5	34	0.019939	1.20E-01	ko00310
**Genetic Information Processing**	Translation	RNA transport	14	171	0.029068	1.61E-01	ko03013
**Metabolism**	Metabolism of cofactors and vitamins	Vitamin B6 metabolism	3	17	0.042643	2.19E-01	ko00750
**Metabolism**	Amino acid metabolism	Tryptophan metabolism	4	30	0.049733	2.32E-01	ko00380
**Metabolism**	Metabolism of other amino acids	Glutathione metabolism	9	103	0.051527	2.32E-01	ko00480
**Metabolism**	Metabolism of terpenoids and	Diterpenoid biosynthesis	3	23	0.090581	3.84E-01	ko00904

	polyketides						
**Genetic Information Processing**	Transcription	RNA polymerase	5	53	0.101214	4.05E-01	ko03020
**Metabolism**	Glycan biosynthesis and metabolism	Other glycan degradation	3	26	0.120251	4.34E-01	ko00511
**Metabolism**	Amino acid metabolism	Phenylalanine metabolism	5	56	0.120688	4.34E-01	ko00360
**Environmental Information Processing**	Signal transduction	Plant hormone signal transduction	16	264	0.17453	5.98E-01	ko04075
**Metabolism**	Carbohydrate metabolism	Starch and sucrose metabolism	12	217	0.319825	1.00E+00	ko00500
**Environmental Information Processing**	Membrane transport	ABC transporters	3	44	0.34132	1.00E+00	ko02010
**Metabolism**	Lipid metabolism	Fatty acid elongation	2	26	0.346714	1.00E+00	ko00062
**Metabolism**	Biosynthesis of other secondary metabolites	Stilbenoid, diarylheptanoid and gingerol biosynthesis	2	27	0.363983	1.00E+00	ko00945
**Metabolism**	Energy metabolism	Nitrogen metabolism	3	46	0.367378	1.00E+00	ko00910
**Metabolism**	Amino acid metabolism	Cysteine and methionine metabolism	6	108	0.396396	1.00E+00	ko00270
**Genetic Information Processing**	Folding, sorting and degradation	SNARE interactions in vesicular transport	2	34	0.478921	1.00E+00	ko04130
**Metabolism**	Biosynthesis of other secondary metabolites	Flavonoid biosynthesis	2	35	0.494309	1.00E+00	ko00941
**Genetic Information Processing**	Folding, sorting and degradation	RNA degradation	6	122	0.513657	1.00E+00	ko03018
**Metabolism**	Lipid metabolism	Arachidonic acid metabolism	1	15	0.514248	1.00E+00	ko00590
**Metabolism**	Lipid metabolism	Linoleic acid metabolism	1	15	0.514248	1.00E+00	ko00591
**Metabolism**	Metabolism of terpenoids and polyketides	Zeatin biosynthesis	1	15	0.514248	1.00E+00	ko00908
**Metabolism**	Glycan biosynthesis and metabolism	Other types of O-glycan biosynthesis	1	17	0.558905	1.00E+00	ko00514
**Metabolism**	Metabolism of other amino acids	Selenocompound metabolism	1	18	0.579676	1.00E+00	ko00450
**Metabolism**	Global and Overview	Degradation of aromatic compounds	1	19	0.599472	1.00E+00	ko01220

**Metabolism**	Metabolism of other amino acids	Cyanoamino acid metabolism	2	45	0.631506	1.00E+00	ko00460
**Metabolism**	Metabolism of cofactors and vitamins	Nicotinate and nicotinamide metabolism	1	23	0.669795	1.00E+00	ko00760
**Metabolism**	Metabolism of cofactors and vitamins	Folate biosynthesis	1	23	0.669795	1.00E+00	ko00790
**Metabolism**	Metabolism of cofactors and vitamins	One carbon pool by folate	1	29	0.752893	1.00E+00	ko00670
**Cellular Processes**	Transport and catabolism	Peroxisome	3	90	0.802379	1.00E+00	ko04146
**Metabolism**	Carbohydrate metabolism	Pentose and glucuronate interconversions	2	65	0.817194	1.00E+00	ko00040
**Metabolism**	Metabolism of cofactors and vitamins	Pantothenate and CoA biosynthesis	1	36	0.823879	1.00E+00	ko00770
**Genetic Information Processing**	Translation	mRNA surveillance pathway	4	121	0.827707	1.00E+00	ko03015
**Metabolism**	Carbohydrate metabolism	Amino sugar and nucleotide sugar metabolism	4	122	0.832523	1.00E+00	ko00520
**Metabolism**	Metabolism of terpenoids and polyketides	Carotenoid biosynthesis	1	41	0.861761	1.00E+00	ko00906
**Metabolism**	Carbohydrate metabolism	Galactose metabolism	2	74	0.869441	1.00E+00	ko00052
**Cellular Processes**	Transport and catabolism	Endocytosis	6	184	0.871086	1.00E+00	ko04144
**Cellular Processes**	Transport and catabolism	Phagosome	2	76	0.879021	1.00E+00	ko04145
**Cellular Processes**	Transport and catabolism	Regulation of autophagy	1	44	0.880471	1.00E+00	ko04140
**Metabolism**	Metabolism of cofactors and vitamins	Ubiquinone and other terpenoid-quinone biosynthesis	1	48	0.901553	1.00E+00	ko00130
**Metabolism**	Lipid metabolism	Sphingolipid metabolism	1	48	0.901553	1.00E+00	ko00600
**Genetic Information Processing**	Folding, sorting and degradation	Ubiquitin mediated proteolysis	4	142	0.907559	1.00E+00	ko04120
**Genetic Information Processing**	Transcription	Basal transcription factors	1	50	0.910661	1.00E+00	ko03022
**Genetic Information Processing**	Transcription	Spliceosome	7	229	0.920345	1.00E+00	ko03040
**Metabolism**	Carbohydrate metabolism	Citrate cycle (TCA cycle)	1	53	0.922773	1.00E+00	ko00020

**Metabolism**	Energy metabolism	Oxidative phosphorylation	5	179	0.929458	1.00E+00	ko00190
**Metabolism**	Lipid metabolism	Fatty acid degradation	1	58	0.939434	1.00E+00	ko00071
**Metabolism**	Amino acid metabolism	Alanine, aspartate and glutamate metabolism	1	58	0.939434	1.00E+00	ko00250
**Genetic Information Processing**	Translation	Aminoacyl-tRNA biosynthesis	1	58	0.939434	1.00E+00	ko00970
**Metabolism**	Lipid metabolism	alpha-Linolenic acid metabolism	1	59	0.942309	1.00E+00	ko00592
**Metabolism**	Metabolism of cofactors and vitamins	Porphyrin and chlorophyll metabolism	1	59	0.942309	1.00E+00	ko00860
**Metabolism**	Amino acid metabolism	Tyrosine metabolism	1	61	0.947658	1.00E+00	ko00350
**Metabolism**	Amino acid metabolism	Glycine, serine and threonine metabolism	1	69	0.964546	1.00E+00	ko00260
**Organismal Systems**	Environmental adaptation	Plant-pathogen interaction	5	210	0.973025	1.00E+00	ko04626
**Metabolism**	Carbohydrate metabolism	Pyruvate metabolism	1	90	0.987287	1.00E+00	ko00620
**Metabolism**	Lipid metabolism	Glycerophospholipid metabolism	1	99	0.99182	1.00E+00	ko00564
**Metabolism**	Carbohydrate metabolism	Glycolysis / Gluconeogenesis	2	151	0.994535	1.00E+00	ko00010
**Genetic Information Processing**	Folding, sorting and degradation	Protein processing in endoplasmic reticulum	4	245	0.997566	1.00E+00	ko04141
**Genetic Information Processing**	Translation	Ribosome	6	384	0.999831	1.00E+00	ko03010
**Metabolism**	Global and Overview	Biosynthesis of amino acids	2	272	0.999978	1.00E+00	ko01230
**Metabolism**	Global and Overview	Carbon metabolism	1	304	1	1.00E+00	ko01200

Table S12 Enriched KEGG pathway analysis of DEGs between IN and RT.

KEGG_A_class	KEGG_B_class	Pathway	IN-VS-RT (1915)	All (5135)	Pvalue	Qvalue	Pathway ID
**Metabolism**	Biosynthesis of other secondary metabolites	Phenylpropanoid biosynthesis	149	196	2.31E-29	2.92E-27	ko00940
**Environmental Information Processing**	Signal transduction	Plant hormone signal transduction	143	264	7.88E-09	4.47E-07	ko04075
**Organismal Systems**	Environmental adaptation	Plant-pathogen interaction	118	210	1.06E-08	4.47E-07	ko04626
**Metabolism**	Energy metabolism	Photosynthesis	51	76	1.07E-07	3.37E-06	ko00195
**Metabolism**	Energy metabolism	Photosynthesis - antenna proteins	22	25	2.17E-07	5.46E-06	ko00196
**Metabolism**	Carbohydrate metabolism	Starch and sucrose metabolism	115	217	1.10E-06	2.32E-05	ko00500
**Metabolism**	Lipid metabolism	Cutin, suberine and wax biosynthesis	21	26	7.04E-06	1.27E-04	ko00073
**Genetic Information Processing**	Replication and repair	DNA replication	39	60	1.11E-05	1.75E-04	ko03030
**Metabolism**	Biosynthesis of other secondary metabolites	Flavonoid biosynthesis	25	35	4.08E-05	5.71E-04	ko00941
**Metabolism**	Metabolism of cofactors and vitamins	Porphyrin and chlorophyll metabolism	37	59	6.01E-05	7.57E-04	ko00860
**Metabolism**	Lipid metabolism	alpha-Linolenic acid metabolism	36	59	0.000171	1.95E-03	ko00592
**Metabolism**	Metabolism of other amino acids	Glutathione metabolism	55	103	0.000565	5.93E-03	ko00480
**Metabolism**	Metabolism of other amino acids	Cyanoamino acid metabolism	27	45	0.001579	1.49E-02	ko00460
**Metabolism**	Metabolism of terpenoids and polyketides	Carotenoid biosynthesis	25	41	0.001699	1.49E-02	ko00906
**Metabolism**	Lipid metabolism	Fatty acid degradation	33	58	0.001778	1.49E-02	ko00071

**Genetic Information Processing**	Replication and repair	Mismatch repair	28	48	0.002356	1.86E-02	ko03430
**Genetic Information Processing**	Replication and repair	Base excision repair	29	52	0.004926	3.37E-02	ko03410
**Metabolism**	Lipid metabolism	Linoleic acid metabolism	11	15	0.004973	3.37E-02	ko00591
**Metabolism**	Amino acid metabolism	Tyrosine metabolism	33	61	0.005297	3.37E-02	ko00350
**Metabolism**	Energy metabolism	Carbon fixation in photosynthetic organisms	51	102	0.005503	3.37E-02	ko00710
**Metabolism**	Global and Overview	Degradation of aromatic compounds	13	19	0.005769	3.37E-02	ko01220
**Metabolism**	Biosynthesis of other secondary metabolites	Stilbenoid, diarylheptanoid and gingerol biosynthesis	17	27	0.005891	3.37E-02	ko00945
**Metabolism**	Amino acid metabolism	Phenylalanine metabolism	30	56	0.009195	4.95E-02	ko00360
**Metabolism**	Amino acid metabolism	Tryptophan metabolism	18	30	0.009429	4.95E-02	ko00380
**Metabolism**	Metabolism of cofactors and vitamins	Ubiquinone and other terpenoid-quinone biosynthesis	26	48	0.012399	6.25E-02	ko00130
**Metabolism**	Energy metabolism	Nitrogen metabolism	25	46	0.013354	6.47E-02	ko00910
**Metabolism**	Biosynthesis of other secondary metabolites	Isoquinoline alkaloid biosynthesis	17	29	0.015642	7.30E-02	ko00950
**Metabolism**	Carbohydrate metabolism	Pentose and glucuronate interconversions	33	65	0.01765	7.94E-02	ko00040
**Metabolism**	Metabolism of other amino acids	Taurine and hypotaurine metabolism	11	17	0.019957	8.41E-02	ko00430
**Metabolism**	Metabolism of terpenoids and polyketides	Zeatin biosynthesis	10	15	0.020029	8.41E-02	ko00908
**Metabolism**	Carbohydrate metabolism	Galactose metabolism	36	74	0.02914	1.18E-01	ko00052

**Metabolism**	Metabolism of terpenoids and polyketides	Diterpenoid biosynthesis	13	23	0.047084	1.81E-01	ko00904
**Metabolism**	Lipid metabolism	Glycerolipid metabolism	30	62	0.047462	1.81E-01	ko00561
**Metabolism**	Lipid metabolism	Glycerophospholipid metabolism	45	99	0.057046	2.11E-01	ko00564
**Metabolism**	Lipid metabolism	Fatty acid elongation	14	26	0.06293	2.27E-01	ko00062
**Metabolism**	Metabolism of other amino acids	beta-Alanine metabolism	20	40	0.0679	2.38E-01	ko00410
**Genetic Information Processing**	Replication and repair	Non-homologous end-joining	7	12	0.114477	3.90E-01	ko03450
**Metabolism**	Metabolism of cofactors and vitamins	Biotin metabolism	15	31	0.137237	4.50E-01	ko00780
**Metabolism**	Metabolism of cofactors and vitamins	Vitamin B6 metabolism	9	17	0.139408	4.50E-01	ko00750
**Metabolism**	Metabolism of cofactors and vitamins	Thiamine metabolism	8	16	0.211642	6.54E-01	ko00730
**Metabolism**	Carbohydrate metabolism	Pentose phosphate pathway	29	68	0.212882	6.54E-01	ko00030
**Metabolism**	Biosynthesis of other secondary metabolites	Tropane, piperidine and pyridine alkaloid biosynthesis	10	21	0.223141	6.69E-01	ko00960
**Metabolism**	Biosynthesis of other secondary metabolites	Monobactam biosynthesis	7	14	0.23646	6.93E-01	ko00261
**Metabolism**	Lipid metabolism	Ether lipid metabolism	13	29	0.255489	7.00E-01	ko00565
**Cellular Processes**	Transport and catabolism	Peroxisome	37	90	0.257636	7.00E-01	ko04146
**Metabolism**	Amino acid metabolism	Arginine and proline metabolism	24	57	0.266198	7.00E-01	ko00330
**Metabolism**	Metabolism of terpenoids and polyketides	Limonene and pinene degradation	3	5	0.271724	7.00E-01	ko00903
**Metabolism**	Glycan biosynthesis and metabolism	Other types of O-glycan biosynthesis	8	17	0.27585	7.00E-01	ko00514

**Metabolism**	Metabolism of terpenoids and polyketides	Brassinosteroid biosynthesis	8	17	0.27585	7.00E-01	ko00905
**Metabolism**	Lipid metabolism	Biosynthesis of unsaturated fatty acids	16	37	0.277844	7.00E-01	ko01040
**Metabolism**	Carbohydrate metabolism	Glycolysis / Gluconeogenesis	60	151	0.291482	7.05E-01	ko00010
**Metabolism**	Amino acid metabolism	Alanine, aspartate and glutamate metabolism	24	58	0.302044	7.05E-01	ko00250
**Genetic Information Processing**	Replication and repair	Homologous recombination	24	58	0.302044	7.05E-01	ko03440
**Metabolism**	Lipid metabolism	Sphingolipid metabolism	20	48	0.312548	7.05E-01	ko00600
**Metabolism**	Metabolism of terpenoids and polyketides	Monoterpenoid biosynthesis	2	3	0.313465	7.05E-01	ko00902
**Metabolism**	Biosynthesis of other secondary metabolites	Flavone and flavonol biosynthesis	2	3	0.313465	7.05E-01	ko00944
**Metabolism**	Nucleotide metabolism	Pyrimidine metabolism	51	129	0.327497	7.24E-01	ko00240
**Metabolism**	Energy metabolism	Sulfur metabolism	17	41	0.343421	7.46E-01	ko00920
**Metabolism**	Metabolism of cofactors and vitamins	Pantothenate and CoA biosynthesis	15	36	0.350692	7.49E-01	ko00770
**Metabolism**	Biosynthesis of other secondary metabolites	Glucosinolate biosynthesis	1	1	0.372931	7.83E-01	ko00966
**Metabolism**	Amino acid metabolism	Lysine biosynthesis	7	16	0.383906	7.93E-01	ko00300
**Organismal Systems**	Environmental adaptation	Circadian rhythm - plant	20	50	0.396684	8.06E-01	ko04712
**Metabolism**	Carbohydrate metabolism	Fructose and mannose metabolism	30	77	0.4225	8.45E-01	ko00051
**Metabolism**	Global and Overview	Carbon metabolism	115	304	0.443342	8.70E-01	ko01200
**Metabolism**	Glycan biosynthesis and metabolism	Glycosphingolipid biosynthesis - globo series	4	9	0.448996	8.70E-01	ko00603

**Metabolism**	Biosynthesis of other secondary metabolites	Anthocyanin biosynthesis	2	4	0.477582	9.10E-01	ko00942
**Metabolism**	Carbohydrate metabolism	Ascorbate and aldarate metabolism	14	36	0.483919	9.10E-01	ko00053
**Genetic Information Processing**	Translation	Aminoacyl-tRNA biosynthesis	22	58	0.509596	9.44E-01	ko00970
**Metabolism**	Amino acid metabolism	Lysine degradation	13	34	0.519432	9.45E-01	ko00310
**Metabolism**	Carbohydrate metabolism	Butanoate metabolism	10	26	0.52481	9.45E-01	ko00650
**Metabolism**	Metabolism of other amino acids	Selenocompound metabolism	7	18	0.532863	9.46E-01	ko00450
**Metabolism**	Amino acid metabolism	Cysteine and methionine metabolism	40	108	0.558846	9.78E-01	ko00270
**Metabolism**	Amino acid metabolism	Valine, leucine and isoleucine biosynthesis	10	27	0.583232	1.00E+00	ko00290
**Genetic Information Processing**	Replication and repair	Nucleotide excision repair	26	71	0.591642	1.00E+00	ko03420
**Metabolism**	Amino acid metabolism	Arginine biosynthesis	14	40	0.674891	1.00E+00	ko00220
**Metabolism**	Carbohydrate metabolism	Glyoxylate and dicarboxylate metabolism	29	82	0.681242	1.00E+00	ko00630
**Metabolism**	Glycan biosynthesis and metabolism	Other glycan degradation	9	26	0.681409	1.00E+00	ko00511
**Metabolism**	Carbohydrate metabolism	C5-Branched dibasic acid metabolism	3	9	0.714089	1.00E+00	ko00660
**Metabolism**	Lipid metabolism	Arachidonic acid metabolism	5	15	0.714563	1.00E+00	ko00590
**Metabolism**	Biosynthesis of other secondary metabolites	Caffeine metabolism	1	3	0.753512	1.00E+00	ko00232
**Metabolism**	Glycan biosynthesis and metabolism	Glycosaminoglycan degradation	6	19	0.771224	1.00E+00	ko00531

**Metabolism**	Metabolism of terpenoids and polyketides	Sesquiterpenoid and triterpenoid biosynthesis	3	10	0.785324	1.00E+00	ko00909
**Metabolism**	Metabolism of terpenoids and polyketides	Terpenoid backbone biosynthesis	17	52	0.796609	1.00E+00	ko00900
**Cellular Processes**	Transport and catabolism	Regulation of autophagy	14	44	0.818127	1.00E+00	ko04140
**Cellular Processes**	Transport and catabolism	Endocytosis	63	184	0.828801	1.00E+00	ko04144
**Metabolism**	Global and Overview	Fatty acid metabolism	24	75	0.859276	1.00E+00	ko01212
**Genetic Information Processing**	Folding, sorting and degradation	SNARE interactions in vesicular transport	10	34	0.872185	1.00E+00	ko04130
**Metabolism**	Metabolism of cofactors and vitamins	Riboflavin metabolism	3	12	0.883916	1.00E+00	ko00740
**Metabolism**	Amino acid metabolism	Histidine metabolism	6	22	0.886046	1.00E+00	ko00340
**Environmental Information Processing**	Membrane transport	ABC transporters	13	44	0.891045	1.00E+00	ko02010
**Metabolism**	Glycan biosynthesis and metabolism	Glycosphingolipid biosynthesis - ganglio series	1	5	0.903156	1.00E+00	ko00604
**Metabolism**	Amino acid metabolism	Valine, leucine and isoleucine degradation	17	57	0.906657	1.00E+00	ko00280
**Metabolism**	Carbohydrate metabolism	Amino sugar and nucleotide sugar metabolism	39	122	0.908681	1.00E+00	ko00520
**Genetic Information Processing**	Folding, sorting and degradation	RNA degradation	39	122	0.908681	1.00E+00	ko03018
**Metabolism**	Metabolism of cofactors and vitamins	Nicotinate and nicotinamide metabolism	6	23	0.911415	1.00E+00	ko00760
**Metabolism**	Global and Overview	Biosynthesis of amino acids	91	272	0.921398	1.00E+00	ko01230
**Metabolism**	Amino acid metabolism	Phenylalanine, tyrosine and	11	40	0.929215	1.00E+00	ko00400

		tryptophan biosynthesis					
**Metabolism**	Global and Overview	2-Oxocarboxylic acid metabolism	18	62	0.933455	1.00E+00	ko01210
**Metabolism**	Lipid metabolism	Synthesis and degradation of ketone bodies	1	6	0.939307	1.00E+00	ko00072
**Metabolism**	Lipid metabolism	Fatty acid biosynthesis	11	41	0.942823	1.00E+00	ko00061
**Metabolism**	Amino acid metabolism	Glycine, serine and threonine metabolism	20	69	0.943033	1.00E+00	ko00260
**Metabolism**	Lipid metabolism	Steroid biosynthesis	7	29	0.955724	1.00E+00	ko00100
**Metabolism**	Carbohydrate metabolism	Pyruvate metabolism	26	90	0.96387	1.00E+00	ko00620
**Genetic Information Processing**	Folding, sorting and degradation	Protein export	14	53	0.965926	1.00E+00	ko03060
**Metabolism**	Metabolism of cofactors and vitamins	One carbon pool by folate	6	29	0.983363	1.00E+00	ko00670
**Metabolism**	Carbohydrate metabolism	Inositol phosphate metabolism	13	55	0.989654	1.00E+00	ko00562
**Genetic Information Processing**	Folding, sorting and degradation	Sulfur relay system	3	21	0.995203	1.00E+00	ko04122
**Metabolism**	Nucleotide metabolism	Purine metabolism	53	185	0.99528	1.00E+00	ko00230
**Genetic Information Processing**	Translation	RNA transport	48	171	0.99614	1.00E+00	ko03013
**Metabolism**	Metabolism of cofactors and vitamins	Folate biosynthesis	3	23	0.99777	1.00E+00	ko00790
**Genetic Information Processing**	Translation	Ribosome biogenesis in eukaryotes	26	108	0.998899	1.00E+00	ko03008
**Genetic Information Processing**	Transcription	RNA polymerase	10	53	0.998958	1.00E+00	ko03020
**Cellular Processes**	Transport and catabolism	Phagosome	15	76	0.999733	1.00E+00	ko04145

**Environmental Information Processing**	Signal transduction	Phosphatidylinositol signaling system	11	62	0.999785	1.00E+00	ko04070
**Metabolism**	Carbohydrate metabolism	Citrate cycle (TCA cycle)	8	53	0.99991	1.00E+00	ko00020
**Genetic Information Processing**	Folding, sorting and degradation	Ubiquitin mediated proteolysis	31	142	0.99998	1.00E+00	ko04120
**Metabolism**	Glycan biosynthesis and metabolism	Glycosylphosphatidylinositol(GPI) -anchor biosynthesis	1	25	0.999992	1.00E+00	ko00563
**Genetic Information Processing**	Folding, sorting and degradation	Protein processing in endoplasmic reticulum	59	245	0.999998	1.00E+00	ko04141
**Genetic Information Processing**	Transcription	Basal transcription factors	5	50	0.999998	1.00E+00	ko03022
**Metabolism**	Carbohydrate metabolism	Propanoate metabolism	2	35	0.999998	1.00E+00	ko00640
**Genetic Information Processing**	Translation	mRNA surveillance pathway	21	121	1	1.00E+00	ko03015
**Metabolism**	Glycan biosynthesis and metabolism	N-Glycan biosynthesis	4	50	1	1.00E+00	ko00510
**Metabolism**	Energy metabolism	Oxidative phosphorylation	33	179	1	1.00E+00	ko00190
**Genetic Information Processing**	Folding, sorting and degradation	Proteasome	2	47	1	1.00E+00	ko03050
**Genetic Information Processing**	Translation	Ribosome	70	384	1	1.00E+00	ko03010
**Genetic Information Processing**	Transcription	Spliceosome	40	229	1	1.00E+00	ko03040

Table S13 Enriched KEGG pathway analysis of DEGs between IN and LF.

KEGG_A_class	KEGG_B_class	Pathway	IN-VS-LF (1811)	All (5135)	Pvalue	Qvalue	Pathway ID
**Metabolism**	Energy metabolism	Photosynthesis	62	76	9.03E-17	1.12E-14	ko00195
**Genetic Information Processing**	Translation	Ribosome biogenesis in eukaryotes	73	108	5.05E-12	3.13E-10	ko03008
**Metabolism**	Energy metabolism	Photosynthesis - antenna proteins	24	25	2.06E-10	8.52E-09	ko00196
**Genetic Information Processing**	Replication and repair	DNA replication	42	60	3.85E-08	1.19E-06	ko03030
**Genetic Information Processing**	Transcription	Spliceosome	116	229	7.48E-07	1.85E-05	ko03040
**Genetic Information Processing**	Translation	RNA transport	90	171	1.71E-06	3.53E-05	ko03013
**Genetic Information Processing**	Replication and repair	Mismatch repair	33	48	2.17E-06	3.85E-05	ko03430
**Metabolism**	Metabolism of cofactors and vitamins	Porphyrin and chlorophyll metabolism	37	59	1.45E-05	2.24E-04	ko00860
**Genetic Information Processing**	Replication and repair	Homologous recombination	35	58	7.90E-05	1.09E-03	ko03440
**Genetic Information Processing**	Translation	mRNA surveillance pathway	62	121	0.000194	2.40E-03	ko03015
**Genetic Information Processing**	Translation	Aminoacyl-tRNA biosynthesis	32	58	0.001431	1.61E-02	ko00970
**Metabolism**	Nucleotide metabolism	Pyrimidine metabolism	62	129	0.001667	1.72E-02	ko00240
**Genetic Information**	Folding, sorting and degradation	RNA degradation	58	122	0.003168	3.02E-02	ko03018

**Processing**							
**Metabolism**	Carbohydrate metabolism	Glyoxylate and dicarboxylate metabolism	41	82	0.004048	3.59E-02	ko00630
**Genetic Information Processing**	Replication and repair	Nucleotide excision repair	36	71	0.005133	4.24E-02	ko03420
**Genetic Information Processing**	Transcription	RNA polymerase	28	53	0.006318	4.90E-02	ko03020
**Metabolism**	Energy metabolism	Carbon fixation in photosynthetic organisms	48	102	0.008747	6.38E-02	ko00710
**Genetic Information Processing**	Replication and repair	Base excision repair	27	52	0.009763	6.73E-02	ko03410
**Metabolism**	Metabolism of terpenoids and polyketides	Carotenoid biosynthesis	22	41	0.011769	7.68E-02	ko00906
**Metabolism**	Biosynthesis of other secondary metabolites	Flavone and flavonol biosynthesis	3	3	0.04382	2.72E-01	ko00944
**Genetic Information Processing**	Folding, sorting and degradation	Ubiquitin mediated proteolysis	60	142	0.047956	2.83E-01	ko04120
**Metabolism**	Amino acid metabolism	Lysine degradation	17	34	0.054451	3.07E-01	ko00310
**Genetic Information Processing**	Folding, sorting and degradation	Proteasome	22	47	0.067377	3.63E-01	ko03050
**Metabolism**	Lipid metabolism	Cutin, suberine and wax biosynthesis	13	26	0.08725	4.34E-01	ko00073
**Genetic Information Processing**	Replication and repair	Non-homologous end-joining	7	12	0.087588	4.34E-01	ko03450
**Metabolism**	Amino acid metabolism	Phenylalanine metabolism	25	56	0.092198	4.40E-01	ko00360
**Metabolism**	Nucleotide metabolism	Purine metabolism	73	185	0.128158	5.89E-01	ko00230

**Environmental Information Processing**	Signal transduction	Plant hormone signal transduction	102	264	0.133754	5.92E-01	ko04075
**Metabolism**	Metabolism of other amino acids	Glutathione metabolism	42	103	0.140823	6.02E-01	ko00480
**Metabolism**	Metabolism of cofactors and vitamins	Vitamin B6 metabolism	8	17	0.21941	8.80E-01	ko00750
**Metabolism**	Biosynthesis of other secondary metabolites	Flavonoid biosynthesis	15	35	0.220044	8.80E-01	ko00941
**Metabolism**	Amino acid metabolism	Tryptophan metabolism	13	30	0.228576	8.86E-01	ko00380
**Metabolism**	Metabolism of terpenoids and polyketides	Zeatin biosynthesis	7	15	0.251991	9.47E-01	ko00908
**Metabolism**	Metabolism of terpenoids and polyketides	Diterpenoid biosynthesis	10	23	0.26776	9.77E-01	ko00904
**Metabolism**	Metabolism of terpenoids and polyketides	Monoterpenoid biosynthesis	2	3	0.285372	1.00E+00	ko00902
**Metabolism**	Amino acid metabolism	Tyrosine metabolism	24	61	0.293109	1.00E+00	ko00350
**Metabolism**	Biosynthesis of other secondary metabolites	Glucosinolate biosynthesis	1	1	0.352678	1.00E+00	ko00966
**Metabolism**	Biosynthesis of other secondary metabolites	Phenylpropanoid biosynthesis	72	196	0.35646	1.00E+00	ko00940
**Metabolism**	Amino acid metabolism	Glycine, serine and threonine metabolism	26	69	0.37966	1.00E+00	ko00260
**Metabolism**	Global and Overview	Carbon metabolism	110	304	0.386762	1.00E+00	ko01200
**Metabolism**	Metabolism of other amino acids	Taurine and hypotaurine metabolism	7	17	0.390142	1.00E+00	ko00430
**Metabolism**	Carbohydrate metabolism	Glycolysis / Gluconeogenesis	55	151	0.411816	1.00E+00	ko00010
**Metabolism**	Lipid metabolism	Sphingolipid metabolism	18	48	0.425654	1.00E+00	ko00600

**Metabolism**	Metabolism of terpenoids and polyketides	Terpenoid backbone biosynthesis	19	52	0.475701	1.00E+00	ko00900
**Metabolism**	Amino acid metabolism	Alanine, aspartate and glutamate metabolism	21	58	0.489745	1.00E+00	ko00250
**Genetic Information Processing**	Folding, sorting and degradation	Protein export	19	53	0.516502	1.00E+00	ko03060
**Metabolism**	Global and Overview	Degradation of aromatic compounds	7	19	0.528816	1.00E+00	ko01220
**Metabolism**	Metabolism of other amino acids	Cyanoamino acid metabolism	16	45	0.540136	1.00E+00	ko00460
**Metabolism**	Carbohydrate metabolism	Pentose phosphate pathway	24	68	0.544147	1.00E+00	ko00030
**Metabolism**	Biosynthesis of other secondary metabolites	Isoquinoline alkaloid biosynthesis	10	29	0.60449	1.00E+00	ko00950
**Metabolism**	Biosynthesis of other secondary metabolites	Tropane, piperidine and pyridine alkaloid biosynthesis	7	21	0.653242	1.00E+00	ko00960
**Metabolism**	Biosynthesis of other secondary metabolites	Stilbenoid, diarylheptanoid and gingerol biosynthesis	9	27	0.65356	1.00E+00	ko00945
**Metabolism**	Lipid metabolism	Arachidonic acid metabolism	5	15	0.65641	1.00E+00	ko00590
**Metabolism**	Lipid metabolism	Linoleic acid metabolism	5	15	0.65641	1.00E+00	ko00591
**Metabolism**	Carbohydrate metabolism	Ascorbate and aldarate metabolism	12	36	0.656767	1.00E+00	ko00053
**Metabolism**	Lipid metabolism	Synthesis and degradation of ketone bodies	2	6	0.686055	1.00E+00	ko00072
**Metabolism**	Metabolism of cofactors and vitamins	Thiamine metabolism	5	16	0.718771	1.00E+00	ko00730
**Metabolism**	Biosynthesis of other secondary metabolites	Caffeine metabolism	1	3	0.728841	1.00E+00	ko00232
**Metabolism**	Lipid metabolism	alpha-Linolenic acid metabolism	19	59	0.733668	1.00E+00	ko00592

**Metabolism**	Metabolism of cofactors and vitamins	Folate biosynthesis	7	23	0.755613	1.00E+00	ko00790
**Metabolism**	Metabolism of cofactors and vitamins	Ubiquinone and other terpenoid-quinone biosynthesis	15	48	0.767205	1.00E+00	ko00130
**Metabolism**	Metabolism of terpenoids and polyketides	Brassinosteroid biosynthesis	5	17	0.772585	1.00E+00	ko00905
**Metabolism**	Carbohydrate metabolism	Inositol phosphate metabolism	17	55	0.793088	1.00E+00	ko00562
**Metabolism**	Energy metabolism	Nitrogen metabolism	14	46	0.799357	1.00E+00	ko00910
**Metabolism**	Amino acid metabolism	Arginine biosynthesis	12	40	0.805548	1.00E+00	ko00220
**Metabolism**	Lipid metabolism	Biosynthesis of unsaturated fatty acids	11	37	0.809482	1.00E+00	ko01040
**Metabolism**	Metabolism of other amino acids	Selenocompound metabolism	5	18	0.818124	1.00E+00	ko00450
**Organismal Systems**	Environmental adaptation	Circadian rhythm - plant	15	50	0.823845	1.00E+00	ko04712
**Metabolism**	Biosynthesis of other secondary metabolites	Anthocyanin biosynthesis	1	4	0.824529	1.00E+00	ko00942
**Metabolism**	Metabolism of cofactors and vitamins	One carbon pool by folate	8	29	0.856879	1.00E+00	ko00670
**Metabolism**	Carbohydrate metabolism	Fructose and mannose metabolism	23	77	0.869163	1.00E+00	ko00051
**Metabolism**	Carbohydrate metabolism	Galactose metabolism	22	74	0.870903	1.00E+00	ko00052
**Metabolism**	Metabolism of cofactors and vitamins	Nicotinate and nicotinamide metabolism	6	23	0.875133	1.00E+00	ko00760
**Metabolism**	Amino acid metabolism	Phenylalanine, tyrosine and tryptophan biosynthesis	11	40	0.886333	1.00E+00	ko00400
**Metabolism**	Metabolism of terpenoids and polyketides	Limonene and pinene degradation	1	5	0.886462	1.00E+00	ko00903
**Genetic Information**	Folding, sorting and degradation	Protein processing in endoplasmic	78	245	0.889448	1.00E+00	ko04141

**Processing**		reticulum					
**Genetic Information Processing**	Transcription	Basal transcription factors	14	50	0.892271	1.00E+00	ko03022
**Environmental Information Processing**	Membrane transport	ABC transporters	12	44	0.900639	1.00E+00	ko02010
**Cellular Processes**	Transport and catabolism	Phagosome	22	76	0.901769	1.00E+00	ko04145
**Metabolism**	Metabolism of terpenoids and polyketides	Sesquiterpenoid and triterpenoid biosynthesis	2	10	0.916907	1.00E+00	ko00909
**Environmental Information Processing**	Signal transduction	Phosphatidylinositol signaling system	17	62	0.926726	1.00E+00	ko04070
**Metabolism**	Lipid metabolism	Fatty acid elongation	6	26	0.938989	1.00E+00	ko00062
**Metabolism**	Amino acid metabolism	Arginine and proline metabolism	15	57	0.94367	1.00E+00	ko00330
**Metabolism**	Metabolism of cofactors and vitamins	Riboflavin metabolism	2	12	0.959365	1.00E+00	ko00740
**Genetic Information Processing**	Folding, sorting and degradation	Sulfur relay system	4	21	0.968941	1.00E+00	ko04122
**Metabolism**	Glycan biosynthesis and metabolism	N-Glycan biosynthesis	12	50	0.969212	1.00E+00	ko00510
**Metabolism**	Lipid metabolism	Steroid biosynthesis	6	29	0.971953	1.00E+00	ko00100
**Metabolism**	Metabolism of other amino acids	beta-Alanine metabolism	9	40	0.972482	1.00E+00	ko00410
**Metabolism**	Amino acid metabolism	Histidine metabolism	4	22	0.9771	1.00E+00	ko00340
**Metabolism**	Carbohydrate metabolism	Butanoate metabolism	5	26	0.977551	1.00E+00	ko00650
**Metabolism**	Glycan biosynthesis and metabolism	Other glycan degradation	5	26	0.977551	1.00E+00	ko00511
**Metabolism**	Lipid metabolism	Glycerolipid metabolism	15	62	0.978254	1.00E+00	ko00561
**Genetic Information**	Folding, sorting and degradation	SNARE interactions in vesicular	7	34	0.979961	1.00E+00	ko04130

**Processing**		transport					
**Metabolism**	Glycan biosynthesis and metabolism	Glycosphingolipid biosynthesis - globo series	1	9	0.98012	1.00E+00	ko00603
**Metabolism**	Biosynthesis of other secondary metabolites	Monobactam biosynthesis	2	14	0.980552	1.00E+00	ko00261
**Metabolism**	Carbohydrate metabolism	Pyruvate metabolism	23	90	0.982149	1.00E+00	ko00620
**Metabolism**	Metabolism of cofactors and vitamins	Biotin metabolism	6	31	0.983764	1.00E+00	ko00780
**Metabolism**	Glycan biosynthesis and metabolism	Glycosaminoglycan degradation	3	19	0.984133	1.00E+00	ko00531
**Metabolism**	Lipid metabolism	Fatty acid degradation	13	58	0.98838	1.00E+00	ko00071
**Metabolism**	Lipid metabolism	Ether lipid metabolism	5	29	0.990759	1.00E+00	ko00565
**Metabolism**	Amino acid metabolism	Lysine biosynthesis	2	16	0.990844	1.00E+00	ko00300
**Metabolism**	Glycan biosynthesis and metabolism	Glycosylphosphatidylinositol(GPI) -anchor biosynthesis	4	25	0.991106	1.00E+00	ko00563
**Metabolism**	Energy metabolism	Sulfur metabolism	8	41	0.991468	1.00E+00	ko00920
**Metabolism**	Amino acid metabolism	Cysteine and methionine metabolism	27	108	0.992138	1.00E+00	ko00270
**Metabolism**	Glycan biosynthesis and metabolism	Other types of O-glycan biosynthesis	2	17	0.99375	1.00E+00	ko00514
**Metabolism**	Carbohydrate metabolism	Starch and sucrose metabolism	60	217	0.994008	1.00E+00	ko00500
**Cellular Processes**	Transport and catabolism	Peroxisome	20	90	0.997585	1.00E+00	ko04146
**Metabolism**	Carbohydrate metabolism	Citrate cycle (TCA cycle)	9	53	0.999086	1.00E+00	ko00020
**Metabolism**	Carbohydrate metabolism	Pentose and glucuronate interconversions	12	65	0.999144	1.00E+00	ko00040
**Metabolism**	Lipid metabolism	Glycerophospholipid metabolism	21	99	0.999265	1.00E+00	ko00564

**Metabolism**	Global and Overview	2-Oxocarboxylic acid metabolism	11	62	0.999324	1.00E+00	ko01210
**Metabolism**	Global and Overview	Biosynthesis of amino acids	72	272	0.999423	1.00E+00	ko01230
**Metabolism**	Global and Overview	Fatty acid metabolism	14	75	0.999535	1.00E+00	ko01212
**Cellular Processes**	Transport and catabolism	Regulation of autophagy	6	44	0.99969	1.00E+00	ko04140
**Metabolism**	Carbohydrate metabolism	Propanoate metabolism	4	35	0.999704	1.00E+00	ko00640
**Metabolism**	Lipid metabolism	Fatty acid biosynthesis	5	41	0.999812	1.00E+00	ko00061
**Metabolism**	Amino acid metabolism	Valine, leucine and isoleucine biosynthesis	2	27	0.999879	1.00E+00	ko00290
**Metabolism**	Carbohydrate metabolism	Amino sugar and nucleotide sugar metabolism	25	122	0.999893	1.00E+00	ko00520
**Organismal Systems**	Environmental adaptation	Plant-pathogen interaction	49	210	0.999949	1.00E+00	ko04626
**Metabolism**	Amino acid metabolism	Valine, leucine and isoleucine degradation	6	57	0.999996	1.00E+00	ko00280
**Metabolism**	Metabolism of cofactors and vitamins	Pantothenate and CoA biosynthesis	2	36	0.999997	1.00E+00	ko00770
**Genetic Information Processing**	Translation	Ribosome	94	384	0.999999	1.00E+00	ko03010
**Metabolism**	Energy metabolism	Oxidative phosphorylation	35	179	0.999999	1.00E+00	ko00190
**Cellular Processes**	Transport and catabolism	Endocytosis	35	184	1	1.00E+00	ko04144

Table S14 Enriched KEGG pathway analysis of DEGs between IN and SC.

KEGG_A_class	KEGG_B_class	Pathway	IN-VS-SC (1100)	All (5135)	Pvalue	Qvalue	Pathway ID
**Genetic Information Processing**	Translation	Ribosome biogenesis in eukaryotes	54	108	3.15E-11	3.85E-09	ko03008
**Genetic Information Processing**	Replication and repair	DNA replication	35	60	4.42E-10	2.70E-08	ko03030
**Metabolism**	Energy metabolism	Photosynthesis	38	76	2.79E-08	9.75E-07	ko00195
**Metabolism**	Biosynthesis of other secondary metabolites	Phenylpropanoid biosynthesis	75	196	3.20E-08	9.75E-07	ko00940
**Metabolism**	Energy metabolism	Photosynthesis - antenna proteins	17	25	6.97E-07	1.70E-05	ko00196
**Metabolism**	Nucleotide metabolism	Pyrimidine metabolism	51	129	1.74E-06	3.54E-05	ko00240
**Genetic Information Processing**	Replication and repair	Mismatch repair	24	48	1.04E-05	1.82E-04	ko03430
**Genetic Information Processing**	Replication and repair	Homologous recombination	27	58	1.60E-05	2.45E-04	ko03440
**Genetic Information Processing**	Replication and repair	Base excision repair	24	52	5.63E-05	7.64E-04	ko03410
**Metabolism**	Biosynthesis of other secondary metabolites	Flavonoid biosynthesis	18	35	8.37E-05	1.02E-03	ko00941
**Genetic Information Processing**	Transcription	Spliceosome	70	229	0.000584	6.47E-03	ko03040
**Metabolism**	Biosynthesis of other secondary metabolites	Stilbenoid, diarylheptanoid and	13	27	0.001806	1.84E-02	ko00945

		gingerol biosynthesis					
**Environmental Information Processing**	Signal transduction	Plant hormone signal transduction	76	264	0.002299	2.16E-02	ko04075
**Genetic Information Processing**	Translation	RNA transport	52	171	0.003244	2.83E-02	ko03013
**Metabolism**	Amino acid metabolism	Phenylalanine metabolism	21	56	0.004216	3.43E-02	ko00360
**Genetic Information Processing**	Transcription	RNA polymerase	19	53	0.010938	7.97E-02	ko03020
**Metabolism**	Nucleotide metabolism	Purine metabolism	53	185	0.011102	7.97E-02	ko00230
**Metabolism**	Amino acid metabolism	Lysine degradation	13	34	0.01874	1.27E-01	ko00310
**Genetic Information Processing**	Replication and repair	Nucleotide excision repair	23	71	0.02025	1.30E-01	ko03420
**Genetic Information Processing**	Replication and repair	Non-homologous end-joining	6	12	0.026725	1.63E-01	ko03450
**Genetic Information Processing**	Folding, sorting and degradation	RNA degradation	35	122	0.033949	1.97E-01	ko03018
**Metabolism**	Biosynthesis of other secondary metabolites	Flavone and flavonol biosynthesis	2	3	0.117949	6.54E-01	ko00944
**Metabolism**	Metabolism of terpenoids and polyketides	Sesquiterpenoid and triterpenoid biosynthesis	4	10	0.147023	7.80E-01	ko00909
**Metabolism**	Amino acid metabolism	Tryptophan metabolism	9	30	0.175376	8.91E-01	ko00380
**Metabolism**	Metabolism of terpenoids and polyketides	Zeatin biosynthesis	5	15	0.202505	9.01E-01	ko00908
**Metabolism**	Biosynthesis of other secondary metabolites	Anthocyanin biosynthesis	2	4	0.202953	9.01E-01	ko00942

**Metabolism**	Metabolism of terpenoids and polyketides	Diterpenoid biosynthesis	7	23	0.20634	9.01E-01	ko00904
**Genetic Information Processing**	Translation	mRNA surveillance pathway	30	121	0.208955	9.01E-01	ko03015
**Metabolism**	Biosynthesis of other secondary metabolites	Glucosinolate biosynthesis	1	1	0.214216	9.01E-01	ko00966
**Metabolism**	Metabolism of other amino acids	Cyanoamino acid metabolism	12	45	0.242862	9.71E-01	ko00460
**Metabolism**	Amino acid metabolism	Alanine, aspartate and glutamate metabolism	15	58	0.246854	9.71E-01	ko00250
**Metabolism**	Glycan biosynthesis and metabolism	Other types of O-glycan biosynthesis	5	17	0.291499	1.00E+00	ko00514
**Metabolism**	Metabolism of terpenoids and polyketides	Limonene and pinene degradation	2	5	0.292037	1.00E+00	ko00903
**Metabolism**	Lipid metabolism	Cutin, suberine and wax biosynthesis	7	26	0.315108	1.00E+00	ko00073
**Metabolism**	Metabolism of other amino acids	beta-Alanine metabolism	10	40	0.347722	1.00E+00	ko00410
**Metabolism**	Carbohydrate metabolism	Starch and sucrose metabolism	49	217	0.361818	1.00E+00	ko00500
**Metabolism**	Metabolism of cofactors and vitamins	Nicotinate and nicotinamide metabolism	6	23	0.368595	1.00E+00	ko00760
**Metabolism**	Metabolism of terpenoids and polyketides	Carotenoid biosynthesis	10	41	0.379441	1.00E+00	ko00906
**Metabolism**	Global and Overview	Degradation of aromatic compounds	5	19	0.385623	1.00E+00	ko01220
**Organismal Systems**	Environmental adaptation	Plant-pathogen	47	210	0.39203	1.00E+00	ko04626

		interaction					
**Metabolism**	Lipid metabolism	Linoleic acid metabolism	4	15	0.405491	1.00E+00	ko00591
**Metabolism**	Amino acid metabolism	Glycine, serine and threonine metabolism	16	69	0.405766	1.00E+00	ko00260
**Metabolism**	Metabolism of other amino acids	Glutathione metabolism	23	103	0.44921	1.00E+00	ko00480
**Metabolism**	Lipid metabolism	Sphingolipid metabolism	11	48	0.456204	1.00E+00	ko00600
**Metabolism**	Lipid metabolism	Fatty acid elongation	6	26	0.494769	1.00E+00	ko00062
**Metabolism**	Metabolism of cofactors and vitamins	Vitamin B6 metabolism	4	17	0.510107	1.00E+00	ko00750
**Metabolism**	Biosynthesis of other secondary metabolites	Caffeine metabolism	1	3	0.51489	1.00E+00	ko00232
**Metabolism**	Metabolism of terpenoids and polyketides	Monoterpenoid biosynthesis	1	3	0.51489	1.00E+00	ko00902
**Metabolism**	Carbohydrate metabolism	Galactose metabolism	16	74	0.529451	1.00E+00	ko00052
**Metabolism**	Carbohydrate metabolism	Pentose and glucuronate interconversions	14	65	0.540092	1.00E+00	ko00040
**Metabolism**	Biosynthesis of other secondary metabolites	Isoquinoline alkaloid biosynthesis	6	29	0.611231	1.00E+00	ko00950
**Genetic Information Processing**	Folding, sorting and degradation	SNARE interactions in vesicular transport	7	34	0.614598	1.00E+00	ko04130
**Metabolism**	Lipid metabolism	alpha-Linolenic acid metabolism	12	59	0.631787	1.00E+00	ko00592
**Metabolism**	Biosynthesis of other secondary metabolites	Tropane, piperidine and pyridine alkaloid biosynthesis	4	21	0.688587	1.00E+00	ko00960
**Metabolism**	Metabolism of cofactors and	Ubiquinone and other	9	48	0.728667	1.00E+00	ko00130

	vitamins	terpenoid-quinone biosynthesis					
**Metabolism**	Amino acid metabolism	Cysteine and methionine metabolism	21	108	0.72941	1.00E+00	ko00270
**Metabolism**	Metabolism of other amino acids	Taurine and hypotaurine metabolism	3	17	0.739105	1.00E+00	ko00430
**Metabolism**	Metabolism of cofactors and vitamins	One carbon pool by folate	5	29	0.775998	1.00E+00	ko00670
**Genetic Information Processing**	Folding, sorting and degradation	Ubiquitin mediated proteolysis	27	142	0.790007	1.00E+00	ko04120
**Metabolism**	Energy metabolism	Nitrogen metabolism	8	46	0.799593	1.00E+00	ko00910
**Metabolism**	Metabolism of cofactors and vitamins	Biotin metabolism	5	31	0.825346	1.00E+00	ko00780
**Metabolism**	Glycan biosynthesis and metabolism	Other glycan degradation	4	26	0.839651	1.00E+00	ko00511
**Metabolism**	Amino acid metabolism	Valine, leucine and isoleucine biosynthesis	4	27	0.861052	1.00E+00	ko00290
**Metabolism**	Lipid metabolism	Arachidonic acid metabolism	2	15	0.863547	1.00E+00	ko00590
**Metabolism**	Carbohydrate metabolism	Glyoxylate and dicarboxylate metabolism	14	82	0.866564	1.00E+00	ko00630
**Metabolism**	Amino acid metabolism	Histidine metabolism	3	22	0.880342	1.00E+00	ko00340
**Metabolism**	Lipid metabolism	Glycerolipid metabolism	10	62	0.883393	1.00E+00	ko00561
**Metabolism**	Glycan biosynthesis and metabolism	Glycosphingolipid biosynthesis - globo series	1	9	0.886002	1.00E+00	ko00603

**Metabolism**	Carbohydrate metabolism	C5-Branched dibasic acid metabolism	1	9	0.886002	1.00E+00	ko00660
**Metabolism**	Metabolism of cofactors and vitamins	Thiamine metabolism	2	16	0.887097	1.00E+00	ko00730
**Metabolism**	Carbohydrate metabolism	Propanoate metabolism	5	35	0.89758	1.00E+00	ko00640
**Metabolism**	Lipid metabolism	Fatty acid biosynthesis	6	41	0.900133	1.00E+00	ko00061
**Metabolism**	Metabolism of terpenoids and polyketides	Brassinosteroid biosynthesis	2	17	0.906829	1.00E+00	ko00905
**Metabolism**	Metabolism of cofactors and vitamins	Pantothenate and CoA biosynthesis	5	36	0.910974	1.00E+00	ko00770
**Metabolism**	Metabolism of other amino acids	Selenocompound metabolism	2	18	0.923297	1.00E+00	ko00450
**Metabolism**	Amino acid metabolism	Tyrosine metabolism	9	61	0.929651	1.00E+00	ko00350
**Metabolism**	Glycan biosynthesis and metabolism	Glycosaminoglycan degradation	2	19	0.936992	1.00E+00	ko00531
**Metabolism**	Carbohydrate metabolism	Butanoate metabolism	3	26	0.939321	1.00E+00	ko00650
**Metabolism**	Lipid metabolism	Steroid biosynthesis	3	29	0.964418	1.00E+00	ko00100
**Metabolism**	Carbohydrate metabolism	Pyruvate metabolism	13	90	0.96558	1.00E+00	ko00620
**Metabolism**	Biosynthesis of other secondary metabolites	Monobactam biosynthesis	1	14	0.965948	1.00E+00	ko00261
**Metabolism**	Carbohydrate metabolism	Ascorbate and aldarate metabolism	4	36	0.966026	1.00E+00	ko00053
**Metabolism**	Carbohydrate metabolism	Inositol phosphate metabolism	7	55	0.96637	1.00E+00	ko00562
**Environmental Information Processing**	Signal transduction	Phosphatidylinositol signaling system	8	62	0.970394	1.00E+00	ko04070

**Metabolism**	Lipid metabolism	Biosynthesis of unsaturated fatty acids	4	37	0.971264	1.00E+00	ko01040
**Genetic Information Processing**	Transcription	Basal transcription factors	6	50	0.971656	1.00E+00	ko03022
**Metabolism**	Metabolism of cofactors and vitamins	Folate biosynthesis	2	23	0.971835	1.00E+00	ko00790
**Environmental Information Processing**	Membrane transport	ABC transporters	5	44	0.973233	1.00E+00	ko02010
**Metabolism**	Amino acid metabolism	Valine, leucine and isoleucine degradation	7	57	0.974636	1.00E+00	ko00280
**Metabolism**	Carbohydrate metabolism	Amino sugar and nucleotide sugar metabolism	18	122	0.977027	1.00E+00	ko00520
**Metabolism**	Amino acid metabolism	Lysine biosynthesis	1	16	0.979007	1.00E+00	ko00300
**Metabolism**	Metabolism of terpenoids and polyketides	Terpenoid backbone biosynthesis	6	52	0.979019	1.00E+00	ko00900
**Metabolism**	Carbohydrate metabolism	Fructose and mannose metabolism	10	77	0.979983	1.00E+00	ko00051
**Metabolism**	Amino acid metabolism	Phenylalanine, tyrosine and tryptophan biosynthesis	4	40	0.982797	1.00E+00	ko00400
**Metabolism**	Energy metabolism	Carbon fixation in photosynthetic organisms	14	102	0.983176	1.00E+00	ko00710
**Metabolism**	Global and Overview	2-Oxocarboxylic acid metabolism	7	62	0.987817	1.00E+00	ko01210
**Metabolism**	Amino acid metabolism	Arginine and proline	6	57	0.990379	1.00E+00	ko00330

		metabolism					
**Cellular Processes**	Transport and catabolism	Regulation of autophagy	4	44	0.99151	1.00E+00	ko04140
**Metabolism**	Lipid metabolism	Ether lipid metabolism	2	29	0.991932	1.00E+00	ko00565
**Genetic Information Processing**	Folding, sorting and degradation	Sulfur relay system	1	21	0.993741	1.00E+00	ko04122
**Metabolism**	Amino acid metabolism	Arginine biosynthesis	3	40	0.995578	1.00E+00	ko00220
**Cellular Processes**	Transport and catabolism	Phagosome	8	76	0.996111	1.00E+00	ko04145
**Metabolism**	Energy metabolism	Sulfur metabolism	3	41	0.99637	1.00E+00	ko00920
**Metabolism**	Lipid metabolism	Fatty acid degradation	5	58	0.997477	1.00E+00	ko00071
**Metabolism**	Lipid metabolism	Glycerophospholipid metabolism	11	99	0.997713	1.00E+00	ko00564
**Metabolism**	Carbohydrate metabolism	Citrate cycle (TCA cycle)	4	53	0.998393	1.00E+00	ko00020
**Metabolism**	Carbohydrate metabolism	Pentose phosphate pathway	6	68	0.99846	1.00E+00	ko00030
**Cellular Processes**	Transport and catabolism	Peroxisome	9	90	0.998761	1.00E+00	ko04146
**Genetic Information Processing**	Translation	Ribosome	60	384	0.998805	1.00E+00	ko03010
**Genetic Information Processing**	Folding, sorting and degradation	Protein processing in endoplasmic reticulum	34	245	0.999233	1.00E+00	ko04141
**Organismal Systems**	Environmental adaptation	Circadian rhythm - plant	3	50	0.999411	1.00E+00	ko04712
**Cellular Processes**	Transport and catabolism	Endocytosis	23	184	0.999472	1.00E+00	ko04144
**Metabolism**	Metabolism of cofactors and vitamins	Porphyrin and chlorophyll metabolism	4	59	0.999494	1.00E+00	ko00860
**Metabolism**	Global and Overview	Fatty acid metabolism	6	75	0.999552	1.00E+00	ko01212
**Metabolism**	Carbohydrate metabolism	Glycolysis / Gluconeogenesis	17	151	0.99968	1.00E+00	ko00010

**Genetic Information Processing**	Translation	Aminoacyl-tRNA biosynthesis	3	58	0.999889	1.00E+00	ko00970
**Metabolism**	Energy metabolism	Oxidative phosphorylation	20	179	0.999909	1.00E+00	ko00190
**Metabolism**	Glycan biosynthesis and metabolism	N-Glycan biosynthesis	2	50	0.999919	1.00E+00	ko00510
**Genetic Information Processing**	Folding, sorting and degradation	Proteasome	1	47	0.999989	1.00E+00	ko03050
**Metabolism**	Global and Overview	Biosynthesis of amino acids	31	272	0.999997	1.00E+00	ko01230
**Genetic Information Processing**	Folding, sorting and degradation	Protein export	1	53	0.999997	1.00E+00	ko03060
**Metabolism**	Global and Overview	Carbon metabolism	33	304	1	1.00E+00	ko01200

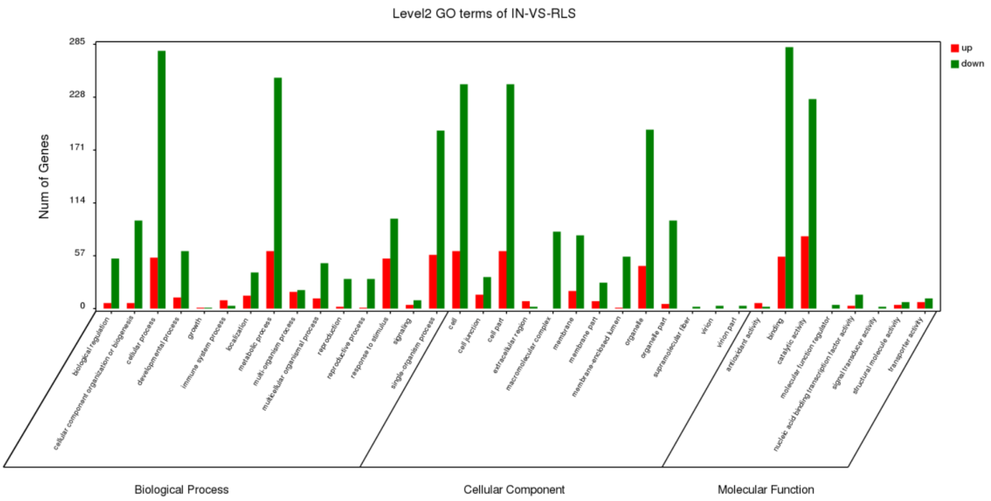

Figure S15 | GO enrichment analysis between the IN and RLS of *A. orientale*.

Tab S16 Primers of the internal control gene and the candidate unigenes used in qRT-PCR.

Gene		Primer 5'--3'	Tm (℃)	Product (bp)
**β-Actin**	F	TGCTGGGTTCAACATCATTCC	60.5	205
	R	CTTGCCCTCAGACTCCTCCT	58.2	
**PIP**	F	CCAACAAGTTCAACGAGAGGC	59.8	178
	R	GTGAGCACGGTGATGTAAAGGA	60.1	
**TIP**	F	TCGTCGCATCCTACCTCCTC	59.9	143
	R	GGTGGCGTAGACGGTGTAGA	58.3	
**NIP**	F	CTTCCACCCGTTCCTGTCTG	60.1	108
	R	GCCGTGACAACAAACAAGAGG	60.5	
**SIP**	F	CTTCTTTGTTAGGACGCTGCTG	59.8	134
	R	ATGTATGGTGGCTTTTGGAGG	59.1	
**DHN**	F	GAGAAGAAGCACGGCGAAAC	60	92
	R	TTCTCATCGCCATCCTTGTG	59	
**C4H**	F	TTGAGGCAAATGGGAATGAT	57.8	90
	R	CCAAGATGGGCAGGGCTA	58.9	
**PAL**	F	GACACCGCCACTTCCATCTT	59.6	174
	R	CTCCTCCCTGACGAACCTGT	58.7	
**TUB**	F	CGGATAACTTCGTGTTCGGTC	59.7	134
	R	TTGGAGGCAGTCGCAGTTC	59.1	
**CYP**	F	ACCCTCCCCTTCTTTACCAAC	59	126
	R	AAACACGATTCCCTTCCCAG	58.9	
**UGD**	F	CCTGACCCACAACAGCAAAG	58.7	125
	R	GTGTCTCCCTACCTCCAATAAGC	59.1	
**SQS**	F	GCTGGATTGGTTGGACTA	60.5	75
	R	AAGAGAATCTGAAGCGAGTT	60.8	
**FPS**	F	CTTGCGTTCTCATAATCTGT	59.7	82
	R	ATCCTGCTTGTGTTGCTA	60.4	
**HMR**	F	AACTGTACCGACCTCAAT	59.3	82
	R	CCTGACTATGCTAGAACCT	59.4	
**MVD**	F	CACTCTGATTCCACTTCTCC	61.5	84
	R	GTCTTGATACTAGCCCTCCT	61.9	
